# Tropical marine sciences: Knowledge production in a web of path dependencies

**DOI:** 10.1371/journal.pone.0228613

**Published:** 2020-02-06

**Authors:** Stefan Partelow, Anna-Katharina Hornidge, Paula Senff, Moritz Stäbler, Achim Schlüter

**Affiliations:** 1 Leibniz Centre for Tropical Marine Research (ZMT), Bremen, Germany; 2 University of Bremen, Bremen, Germany; 3 Jacobs University, Bremen, Germany; Universidad Rey Juan Carlos, SPAIN

## Abstract

Scientific agenda setting is critical at all levels of research, but can be strongly influenced by structural path dependencies of the science system itself. In this article we examine how knowledge production is shaped by interconnected path dependencies using the field of tropical marine sciences as a global case study. We use scientometric analysis methods on an original data set of 1328 peer-reviewed journal publications to examine publication trends including a co-authorship network analysis, links between author origin and research locations as well as a quantitative analysis of terminology use over space (i.e., region) and time. Scientometric findings are analytically discussed through a conceptual framework premised on theories of path dependency. Findings and critical analysis highlight how tropical marine science provides a prominent global example of how North American, European and Australian science programs predominantly shape knowledge production of the global science system, generating critical reflection on the path dependencies these create on current and likely future knowledge production and science agendas. Similar dependencies face other fields of science, and thus this study provides broadly relevant quantitative observational empirical findings supplemented with a critical social science analysis of how a transcultural Science and Technology Studies lens is useful for unpacking the webs of path dependencies driving, inhibiting and/ or shaping global knowledge production, placing meaning and context over observed empirical trends.

## Introduction

How and why do scientists and science organizations choose to do research on certain topics and places? It is evident that there are many reasons, and those reasons may differ at various levels of research organization; be it the individual researcher, the working group, the institute, the university, the state/ country, or the region. Scientific agendas and knowledge production broadly are often perceived to be driven by performance oriented structures of the science systems at hand as well as agency in choice, such as being driven by career and personal interest, the most pressing questions in a discipline or field, the urgency of needed knowledge, or to advance the state-of-the-art in objective ways (e.g., part of the logic for tenured positions and publically funded universities).

However, as is evident in existing scientometric analyses, scientific agendas and outputs at a more foundational level are in large part driven by myriad forms of structural impediments [1–3]. Theoretically, we hypothesize these impediments as path dependencies, at different levels of scientific organization using data analyzed with mixed scientometric methods including network maps to present our descriptive data [4]. Path dependencies can be both latent or more explicit and transparent; in either case they are nonetheless structural pillars driving current scientific agendas, often to a larger extent than is currently reflected on or recognized within specific fields.

This article examines structural path dependencies of the science system that substantially co-determine scientific knowledge production, as well as the implicit science-based human interactions with nature, by empirically focusing on tropical marine sciences as a field of scientific knowledge production. The field of tropical marine sciences is a unique case study for examining how past and current research agendas have been influenced by a number of very influential and historically embedded path dependencies. The field is unique in that its scientific practice is bounded to, and dependent on, specific geographical locations and geopolitical relationships with tropical countries. Many countries which are heavily engaged in tropical marine sciences are not located in, or do not have domestic access to the tropical world, including many North American and European programs. In contrast, many tropical countries have marine and coastal systems of substantial research interest worldwide, but have limited domestic research capacity, infrastructures and funding. In addition, historical relationships between tropical and non-tropical countries are often associated to discourses on North-South or developed-developing country relations, or the impact of prior colonial relations [5].

As such, the 2017 Global Ocean Science Report by UNESCO-IOC [6] assesses: “The USA has the highest number of research institutions varying in size (315)—roughly equal to the total number of research institutions in Europe combined and greatly exceeding the number of institutions operated in Asia and Africa”. Amongst the five largest Ocean science budgets in terms of percentage of national research and development funding, the report identifies those by the USA, Australia, Germany, France and the Republic of Korea (27). Of the overall 784 marine field stations counted by the report nevertheless, 46% are located in Asia (23%), Africa (8%), South America (10%) and Oceania (5%), as well as 54% in Europe (22%), North America (21%), and Antarctica (11%)[6]. In addition, tropical marine sciences are not bound to specific disciplines, but instead span the disciplinary range from natural to social sciences [7–9] with the common research objective of understanding coastal ecosystems, their functioning, use and management in the tropics, acting as a defining and uniting frame.

Tropical marine sciences are (a) highly technology dependent, expensive sciences with, as a consequence, substantial involvement of financing states in agenda setting processes [6,10–12], (b) international with strong historical/ partly colonial roots [13–17], (c) interdisciplinary [8,9,18], and (d) driven by a visible normative orientation and legitimation towards a *sustainable use and management* of tropical coastal zones and their resources [19, 20]. These particularities suggest that an assessment of the field from a transcultural and postcolonial lens is a useful analytical perspective. Such an analysis draws on Science and Technology Studies with the aim to empirically understand the path dependencies guiding research practices, and in turn those being reproduced by them. It is a substantially understudied field, particularly in combination with scientometric data with a global scope, that is nevertheless pertinent for allowing thorough reflection of research practices and forms of international cooperation and engagement influencing how knowledge related to sustainability is produced and utilized. The following scientometrical data therefore aims to fill this gap and encourage discussion on the disciplinary, thematic, regional, historical and geopolitical specificities of scientific knowledge production within tropical marine sciences–and how these data can be interpreted within an increasingly globalized and politicized science scape for this field and others.

### Scientometric analysis to examine global science systems

Bibliographic information are some of the most important data for studying the science system through its primary output of science publications. Analyzing publication trends on research locations, yearly outputs, country distributions, co-authorship and other forms of collaboration [21] that are able to be extracted from these data can reveal important insights about global structural drivers of scientific knowledge production [22–25]. In particular, network analysis has been instrumental in analyzing the distribution of scientific knowledge production globally at the country level [26], showing the dominance of North American and European science outputs, even on topics where much of that knowledge is extracted elsewhere such as in global health research [27], disaster research [24], and land use in agriculture [25]. Scientometric analysis also allows for examining collaboration (e.g., co-authorship) networks overtime, to see proportional changes between domestic and international collaborations as science becomes more interconnected globally [28–30].

In the broader field of marine science, using scientometric data has been employed in sub-fields such as fisheries science [30], marine microplastics [31], sea turtle research [32], estuaries and coasts [33], and marine biodiversity research [34]. Many of these studies include author network analyses, which have been supported by more local and qualitative analyses of scientific collaboration in specific regions, universities or departments [35–37]. Local case studies support the formation of more grounded social theories for analyzing the global science system. For example, through examining the sociology of knowledge production within science and technology studies. This provides context, and thus a way to interpret what the patterns in global level collaborations analyzed via descriptive scientometrics might mean in practice.

Increased scientific collaboration, particularly internationally, has been observed via network analysis and scientometrics as the new reality of the science system [21] and specifically marine science [23,38,39]. One important question is whose reality is this, and who benefits? It is clear that certain regions and countries dominate the advancement of cooperative global knowledge production in science, both driving (e.g., via funding and political mandates) and benefiting from collaboration. Scientometric analysis provides a powerful data-driven lens to observe and descriptively analyze scientific outputs. However, it can be difficult to interpret the broader meaning and context of observational scientometric data to which it applies to for specific people and places within the science system. To begin unpacking this layer of understanding, it is useful to draw on established social theory (e.g., science and technology studies; path dependency; sociology of knowledge) which have been grounded in layers of case studies, to place context and meaning over observed global trends. To our knowledge, this provides a first step towards the novel combination of analyses in this research which has not yet been put forth in the literature, even to our knowledge outside the marine sciences. We first outline our theoretical lens of path dependency below, and then present our scientometric empirical data. In our discussion, we can begin to frame and interpret what these data could mean, suggesting that future work can combine both approaches empirically as next steps.

### Path dependency

Multiple definitions of ‘path dependency’ as a concept exist. An often employed, precise and general enough definition is one by Mahoney [40], understanding path dependencies as past or historical events that set in motion, limit, structure or place deterministic properties on current and future events. Perhaps more simply, path dependency is the dependency of future system states on the past. A critical aspect of path dependency is that system states always have a history. Leaving or deviating from an institutional path (e.g., technological, organizational or discursive) is commonly cost- and resource intensive [41,42]. Following a particular path comes with increasing returns, such that the probability of continuing the same path increases with each move forward [42]. Path dependencies can take numerous forms (Table 1), influencing knowledge production in both direct and indirect ways [43]. Here the scientometric data can be examined from a path dependency perspective, which suggests a hypothesis that the field of tropical marine science is a prominent global example of how historical geopolitical relationships manifest into research outputs today.

**Table 1:** Different types of path dependencies presented as a conceptual framework.

Type of path dependency	Definition/ explanation
(1) Material science infrastructures	Equipment, labs, and access to research vessels and marine research stations are essential for data collection and analysis. Their quality/standard in a particular science system forms a crucial path dependency that plays out in scientific activity in the present.
(2) Immaterial science infrastructures	Here we distinguish access to funding and donor landscapes, language of research and teaching, existing partnership networks and the disciplinary versus theme-oriented (or other) organization of a particular science system. These four are detailed out in the following.
(2a) Access to funding and donor landscape	Where money comes from, what it is supposed to be used for and how much is available shapes dependencies. If the European Union for instance, allocates its research budget to climate change adaptation in Europe, it may be difficult to get EU funding for gender-related research in West Africa. Existing funding lines can be closely tied to historical developments and politics. The global differences in terms of public Research and Development expenditures (as % of GDP) are published on a yearly basis by the UNESCO Institute for Statistics [44].
(2b) Language of research and teaching	Publishing and speaking languages create strong path dependencies. English is the dominant international publishing language, determining how science is communicated and who is advantaged in doing so. The general language spoken in a country can create both challenges and opportunities for research and collaboration from and within that country.
(2c) Science networks—Transregional, national and international	Science collaboration, friendship, grown professional or cultural networks can create dependencies. Educational networks (e.g., place of degree attained) create networks over various generations. Language and funding networks (e.g., mandated consortiums) can be initiators of network development.
(2d) Disciplinary, thematic and type of research-related organization of the science system	Organizing scientific knowledge production by disciplines, thematic foci, or problem orientation has long-term consequences for the type of research done. Degrees of applied vs. basic research, theoretical embedding, philosophies of science and separation or reciprocal co-shaping of theological and scientific outlooks on reality vary and determine the possibilities for the type of future research done within a particular science system.

Drawing on path dependencies through a transcultural Science and Technology Studies lens, broadens the notion of sustainability as a normative social concept, and how we can move society towards it, by recognizing the structural and institutional drivers of the science system itself as an underlying factor. We do not only want to know about the links between social and environmental change, and the normative preferences for addressing them, but how the science system itself is influenced, shaped and in part path dependent on the nexus between historical geopolitical relations and transregional networks. The implications of this hypothesis would suggest a strong influence on how scientific agendas are shaped as well as how knowledge is produced and utilized globally.

In this article we examine publication data with scientometric methods to explore the hypothesis that knowledge production has been and will likely continue to be in part driven by path dependencies using the field of tropical marine sciences as a global case study. We have generated an original data set and conducted numerous scientometric analyses on these data, including general publication trends, co-authorship networks, disciplinary composition, links between author origins and research locations, as well as a quantitative analysis of terminology in publications over space and time. We analyze our findings through a critical social theoretical lens of transcultural Science and Technology Studies, drawing on the concept of path dependencies in our discussion to demonstrate how these two different analysis methods with different disciplinary origins can be combined for future joint empirical analyses to advance the understanding and impact of knowledge production trends in different fields of study. The field of tropical marine sciences likely represents similar path dependencies facing other fields, and thus provides a relevant multi-/ inter-disciplinary case example for more generalizable reflections on broader knowledge production trends in science.

## Methods

Data used in this analysis were compiled by the authors through a literature review at the global level from 1979–2018 [9]. We identified 2877 peer-reviewed English language publications of potential relevance within the SCOPUS database, within all journals contained in the database, using the following query on May 15, 2019: [(TITLE-ABS-KEY ((tropic* OR coral OR equator*) AND (marine OR ocean OR sea) AND (coast* OR shore OR intertidal)) AND TITLE-ABS-KEY ((social OR ecological) OR ses OR socio OR economic OR institution* OR govern* OR management OR biophy*) AND TITLE-ABS-KEY (component* OR attribute* OR dynamic* OR drivers OR challenge* OR sustainab* OR communit* OR fisher* OR case* OR site OR service* OR (marine protected area* OR mpa)) ]. 96 non-English language publications were identified in the initial search. However, we excluded non-English language publications for consistency in the qualitative data analysis and due to a lack of language expertise in coding the many languages. Based on our original search sample size and results, we estimate that non-English language publications only represent ~3.3% of the total sample, when considering our results presented below. The initial resulting publications were scoped down to 1328 publications for use through a process of reviewing all titles and abstracts individually for fit to the study scope (S4 Table). The final publications (those within the scope) were limited to the publications in English, as main language of international scientific exchange (exceeding Russian, Spanish and Chinese in internationally accessible scientific journal publications produced [45]) related to social, ecological, or social–ecological research in the marine and coastal tropics. The ‘tropics’ refers to research focused on geographical locations or context between latitudes 23.5 N and 23.5 S. Publications could relate to the marine and coastal context through a wide variety of topics ranging from land-based social research to exclusively marine natural system processes or land–sea connectivity across any scientific discipline [9]. The temporal range of the sample or the journals published in were not purposively restricted beyond the limitations of the database itself. Publications range from 1979–2018. Categories of data collected are shown in Table 2. Data was not collected on funding sources due to a lack of sufficient and reliable data from each paper in the sample.

**Table 2:** Review categories and definitions used in this analysis.

Category	Definition
Author origin	Country of author stated on publication affiliation. If multiple in different countries, the first was taken.
Research location	Regional location of empirical focus of the research taken from the abstract or full text if needed. If necessary, multiple locations per article were used.
Number of authors per article	The number of authors in the authorship list of the publication.
Publication title and abstract	The title and abstract text of each publication was used for qualitative data analysis.
Year	Year publication was published.
Domestic collaboration	Two authors on the same publication from the same countries in their affiliation.
International collaboration	Two authors on the same publication from different countries.
Single author	Only one author on the publication.
Publication disciplinary focus	Based on classification of social, ecological or social-ecological processes focused on in each publication, based on Partelow et al., (2018) review categories.

### Data analysis

#### Authorship and author origin

Author origin and author location were combined to analyze the number of single author vs. multi-author publications in each year. Multi-author publications were split into two categories, domestic and international collaborations. Domestic collaborations only have authors from the same country. International collaborations have authors from multiple countries. In addition, the number of authors per country was calculated to compare the ratio and test hypotheses that certain countries may have more authors, and that the ratio between first authors and non-first authors in a country may differ. Each publication was further categorized into the main discipline(s) of the research. The composition of disciplinary contributions in the data were examined with basic statistics. Both the origin of the first-author (by continent) and the location of the research study (by region) were conducted over time in five-year intervals.

#### Co-authorship trends

The ratio of domestic and international co-authors connections was calculated from each publication in the sample, aggregated at the country level, and a ratio was calculated for each country irrespective of the total number per country. The descriptive statistics of the total number of all co-authors per country was calculated along with the number of countries each country has co-authored publications in the sample with. A univariate linear regression model was run between the domestic/interactional ratio data and the total number of co-authors data at the country level. To correct for heteroscedasticity, the total number of co-authors was log transformed to be recalculated in the model (S3 Fig for model diagnostics).

#### Undirected network analysis of author collaborations

Social network analyses of co-authorship was performed in the igraph package of the R Core statistical program [46]. Authors were assigned to the country (i.e., nodes in the network) of their affiliation provided on the publication. The first affiliation was used for consistency if multiple existed. An undirected network analysis (i.e., the direction of the connection between nodes is non-informative) of co-authorship was generated between all authors of all publications, regardless of authorship order (i.e., first or last author). Network and node level metrics were used within the iGraph package to analyze centrality measures (i.e., eigenvector centrality, strength, betweenness, closeness, degree) and network size (i.e., diameter, mean distance) (S6 Table).

#### Directed network analysis of research locations

Publications were categorized by author, but also by the geographical location or focus of the research in the tropics. Research region was determined by individually coding each abstract (and referencing the full text if necessary for clarification) on the geographical location where the research was being conducted using the same regional classification scheme as above. If the paper did not specify a region, it was coded as non-specific, and later excluded from the network calculation. A directed network analysis was conducted (i.e., the connection between nodes is directional and informative) with an arrow going from the origin country of the first author to the region of the research location or context. Country level was used for author origin, but regional level was used for research location to aggregate totals to identify more prominent regional trends. Only first author origins were used, as they are representative of the broader sample of all authors (S1 Table).

#### Terminological cluster analysis

An analysis examining clusters of used terminology in publications over space and time was conducted using a quantitative approach to qualitative data analysis. Publication title and abstracts of publications were used as data, and data collection and formatting was conducted using the qualitative data analysis software MaxQDA [47]. Individual words were extracted from each title and abstract. A quantitative presence/ absence data set of word occurrence across the publication data was exported from MaxQDA. Subsequent data analysis was performed in R [48].

To identify assemblages of words that represent different terminologies, we ran a hierarchical cluster analysis in *R-mode*. The abundances of each word per abstract were converted to presence/ absence data. Words that contributed less than 0.1% to the overall count of all words were removed from the dataset, narrowing down the number of words from 10418 to 186. Subsequently, words deemed non-informative were removed and synonyms united (i.e., lemmatization; c.f., S3 Table), resulting in a final dataset of 114 indicator words in 749 abstracts, with 78.4% zeroes (i.e., non-observations).

Clustering was based on an Ochiai dissimilarity matrix. Complete linkage agglomerative clustering produced the best interpretable dendrogram, and was thus chosen as our linkage method of choice, despite the single linkage and average agglomerative clustering (UPGMA) resulting in higher correlations of the respective dendrograms’ cophenetic distances with the underlying distance matrix.

The selection of the optimum number of clusters was based on comparisons between binary matrices of the dendrogram cut at different levels, and the original Ochiai dissimilarity matrix. The comparison was performed as a Pearson’s correlation between both matrices [49]. As we were interested in holding the final number of clusters fairly low for the sake of interpretability, we monitored the change in the correlation between distance matrix and binary matrix for every increase in the number of clusters tested (k). Having 2 instead of 1 cluster resulted in the largest increase in correlation of both (S1 Fig), but since clustering with only 2 groups is barely informative, we chose the second optimum of 6 groups as our final partitioning. S2 Fig displays these clusters, which we considered as different terminology clusters, in a dendrogram.

To illustrate the progression of the six terminology clusters through time and space, and their annual contribution to the overall literature on tropical marine sciences, we took the summed contribution of each terminology (i.e., the number of occurrences of its keywords in the abstracts of each year) and divided this sum by the total number of indicator words occurring in that year (Fig 6; mid). We then addressed the spatial dimension of the path dependencies of the six terminology clusters by assigning each abstract to the origin of the first author. Countries were aggregated into World Bank regions (World Bank, 2015) and, for each cluster, the contribution of each region to that cluster was calculated (Fig 6; bottom).

Fig 6 (top) illustrates, for each year and region, in how far the clusters’ occurrences deviate from the degree that would be expected if indicator words were uniformly distributed across the six clusters and regions in each year. White blocks indicate the region and terminology to contribute to the year’s body of indicator words as to be expected if words were even across terminologies, space and time. Shades of brown represents positive anomalies, while turquoise ones are negative. Prior to plotting, the anomalies were square root transformed, with the sign function maintained. This transformation reduces the importance of extreme values while raising that of intermediate ones. This assists visual inspection of the heat-map through broadening the spectrum of shades of turquoise and brown displayed, rather than showing a few extreme values only with a vast indifferent majority of white blocks.

## Results

### Publication trends

**Fig 1:**
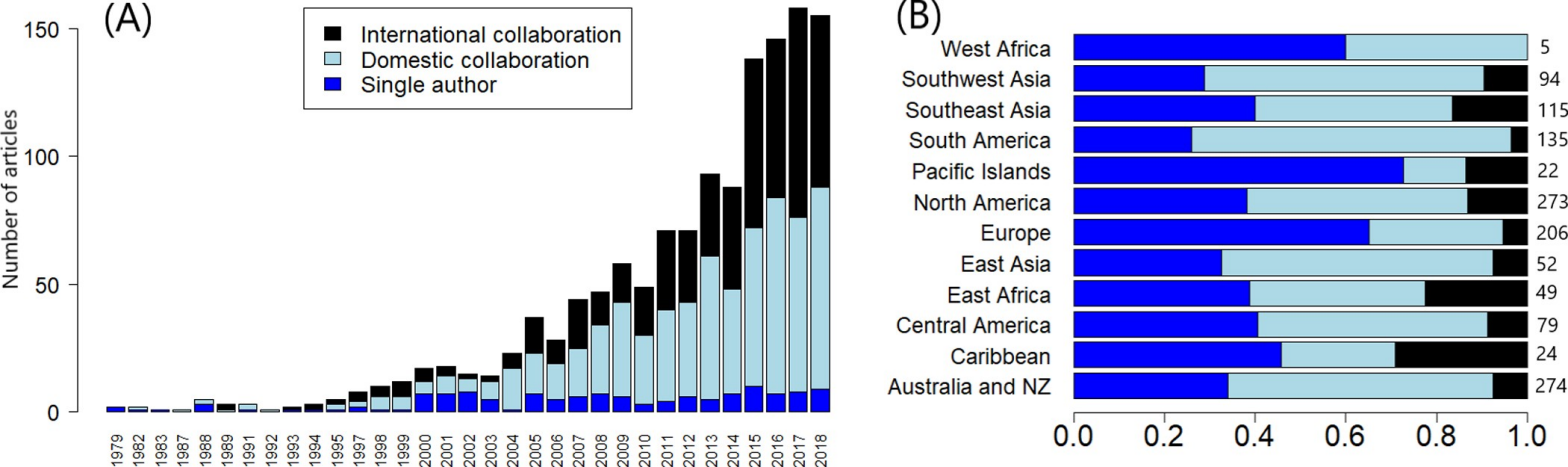
(A) The number of publications in tropical marine science over time. Each year is subdivided into the type of publication, either single author or multi-author. Multi-author publications are referred to as collaboration publications, if all authors are from the same country they are classified as domestic and if there are authors from different countries they are classified as international. (B) Proportion of single author and collaboration publications by the region of the first author. Total N is shown to the right.

Co-authorship trends are changing over time. Publications per year are increasing, and the percentage collaborative publications is driving this growth (Fig 1). This follows similar trends in related fields [50]. However, growth is not even across space or time, nor is it even within collaborative publications, with increasing percentages of domestic collaborations over time. International collaborations are increasing but are still at lower percentages compared to domestic collaborations. Observed trends suggest that international collaborations will likely make up a larger portion of future publications. Publications with European, North American and Australian first authors have the highest percentages of collaborative publications, and the lowest single author percentages. These regions lead in the total amount of published publications, indicated by first author (Fig 1B and Tables S1 and 3). Data on the percentages of different disciplinary contributions and ratios are not even, and show with data until 2014 in S2 Table.

**Table 3:** Total number of first author and total authors per country. Only countries with more than 1%, all other countries are below 1% in both percentage lead author and all authors. Full table in S1 Table.

Country	Percentage lead author	Total N lead author	Percentage all authors	Total N all authors
Australia	20.26	269	20.03	1210
USA	18.07	240	17.48	1056
Brazil	8.21	109	8.16	493
UK	4.89	65	4.55	275
India	3.99	53	3.18	192
Mexico	3.99	53	3.69	223
France	3.09	41	3.66	221
Philippines	2.71	36	2.80	169
Germany	2.64	35	2.65	160
Canada	2.48	33	1.94	117
China	2.18	29	2.33	141
Malaysia	1.81	24	1.79	108
Kenya	1.20	16	1.22	74
Costa Rica	1.13	15	1.09	66
Indonesia	1.13	15	1.69	102
Singapore	1.13	15	1.41	85
Sweden	1.13	15	1.11	67

**Fig 2:**
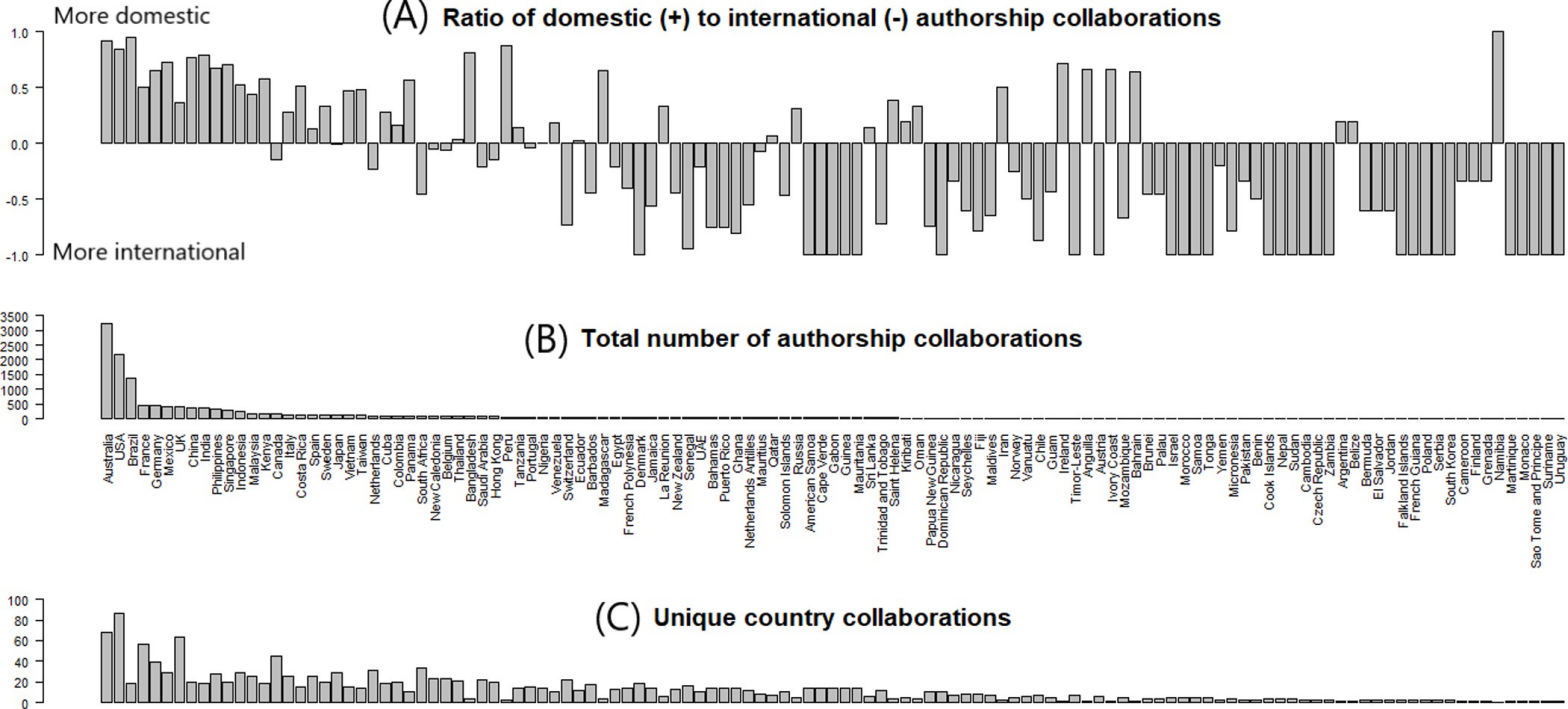
Comparison of co-authorship trends at the country level. (A) Ratio of the number of domestic vs international co-authors per country. (B) Total number of co-authors per country. (C) Number of countries collaborated with (at least once).

The ratio a country has between the number of domestic vs international co-authors is shown in Fig 2 (Also see S4 Fig for scatterplots). The first ten countries in all three graphs, from left to right, are: Australia, USA, Brazil, France, Germany, Mexico, UK, China, India and the Philippines. As the number of co-authors increases, and the number of collaborators from different countries increases, the higher the percentage of domestic collaborations a country will have in its co-authorship networks. A linear regression model between the ratio of domestic vs international co-authors (Fig 2A) and the log transformed data for total number of co-authors (Fig 2B) is highly significant with a p-value of 0.01e-13 and an adjusted R-squared of 0.4113.

### Author origin and collaboration networks

Table 3 shows the total number of lead authors and total authors from each country. Australia and the USA are the most dominant countries as both lead and total number of authors. In general, when comparing countries, the percentage of lead authors matches the percentage of all authors from that country (Table 3). The top four countries (Australia, USA, Brazil, UK) contribute more than 50% of all authors, and the top 10 countries contribute 70% of all authors (S1 Table). There are no Caribbean or West African countries with significant amounts of contributing authors, a few Southeast Asian countries and one Central American country (Costa Rica) with noticeable relative authorship contributions. The relative proportion of authors from each time interval is relatively stable, with the exception that African authorship has not increased proportionally over the last 10 years. Many of the countries in the top 20 contributing authors (S1 Table) do not have domestic tropical coasts (e.g., Canada, Sweden), some have current territories (e.g., France, USA) and some have former territories or colonies (e.g., UK, France, Germany, Spain). However, many, if not most tropical countries, contribute relatively minimal authors to tropical marine science English language publications.

**Fig 3:**
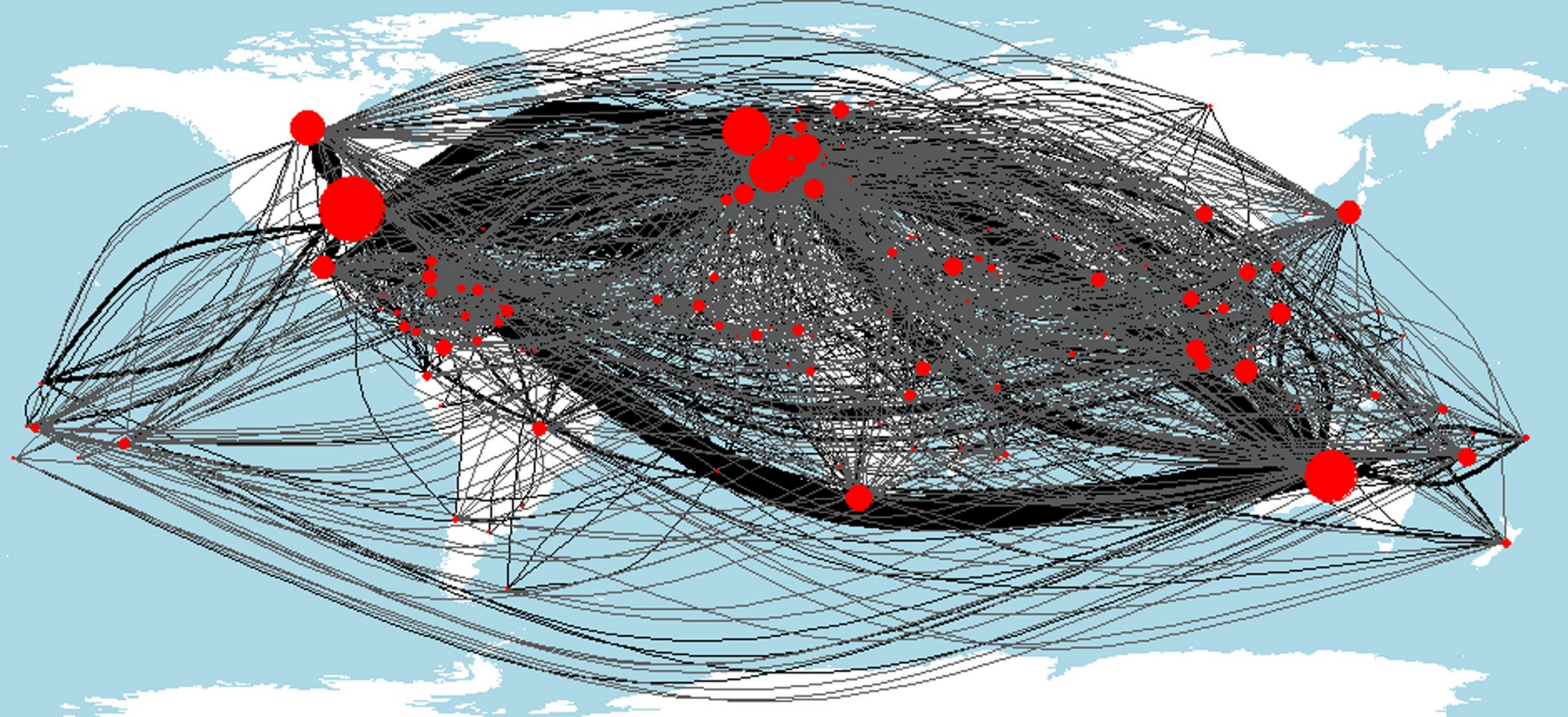
Spatial projection of an undirected network analysis of international authorship collaboration between countries. Red circular nodes indicate the number of international collaborations of individual countries. Node size is dependent on the total number of collaborations (i.e., all incoming and outgoing edge lines). The thickness of edge lines between countries represents the number of publications with authors from both countries. Node and edges sizes are scaled differently to enable better visualization.

Between country authorship collaborations, both domestic and international, are shown in Fig 3. Three pieces of information are shown. First, the connection between countries, where the thickness of the line (i.e., edge connection) between countries indicates the number of publications with authors from both countries. Second, the size and color of the node (i.e., the circle over each country). The red circles represent the total number of collaborations with authors from the country. In addition, the number of unique international collaborations per country is shown in Fig 2C and S7 Table.

Western/ Northern countries are the most collaborative with strong links between other Western/ Northern countries and Australia. However, Western/ Northern countries do not cooperate evenly across tropical countries. Australia, Brazil and USA have larger relative proportions of domestic collaborations. Few international collaborations exist between tropical countries, at least proportionally to Western/Northern international collaborations dominated by Europe, North America and Australia. Despite all research being geographically located or focused in the tropics, most of the research is published by authors outside tropical regions, and most of the collaborations within this research are between authors from Western/ Northern countries.

Node centrality metrics (Table 4) were calculated for the undirected authorship network (Fig 3) including eigenvector centrality, strength, betweenness, closeness and degree (S5 Table). Node centrality measures indicate that, generally, the USA and Australia play central roles. We can see that some countries have a relatively low total number of authors, but they are connected with a diversity of authors from different countries, such as Indonesia, the Philippines, Malaysia, Canada and New Caledonia. Countries such as Mexico and Brazil have higher total authorship but lower diversity of connections to author from other countries. Additional network metrics were calculated for the undirected authorship network (Fig 3) indicating the diameter of the network is 4 (the maximum distance away from authors in another country) with a mean distance of 2.183 (the average distance away from authors in another country). Definitions of how each network metric is calculated are provided in S6 Table.

**Table 4:** Node centrality metrics for each network analysis. Only the top 10 countries (nodes) are shown for undirected authorship network analysis. All twelve regions are shown in the directed author-to-region network. Full lists for undirected authorship network analysis can be found in S5 and S6 Tables.

Undirected international authorship network						Directed author-to-region network	
*Degree*		*Strength*		*Eigenvector centrality*		*In-degree (unweighted)*	
USA	172	USA	5286	USA	1.000	Southeast Asia	28
Australia	136	Australia	3416	Australia	0.913	Southwest Asia	28
UK	126	UK	2232	UK	0.631	East Africa	28
France	112	France	1506	France	0.320	Pacific Islands	26
Canada	90	Germany	1050	Canada	0.306	Caribbean	23
Germany	78	Canada	860	Philippines	0.288	South America	18
South Africa	66	Philippines	844	Germany	0.277	Central America	16
Netherlands	62	Mexico	782	Namibia	0.241	East Asia	11
Indonesia	58	Malaysia	736	Malaysia	0.234	Australia and NZ	11
Japan	58	Indonesia	678	Mexico	0.225	Middle Africa	8

### Networks of research locations

**Fig 4:**
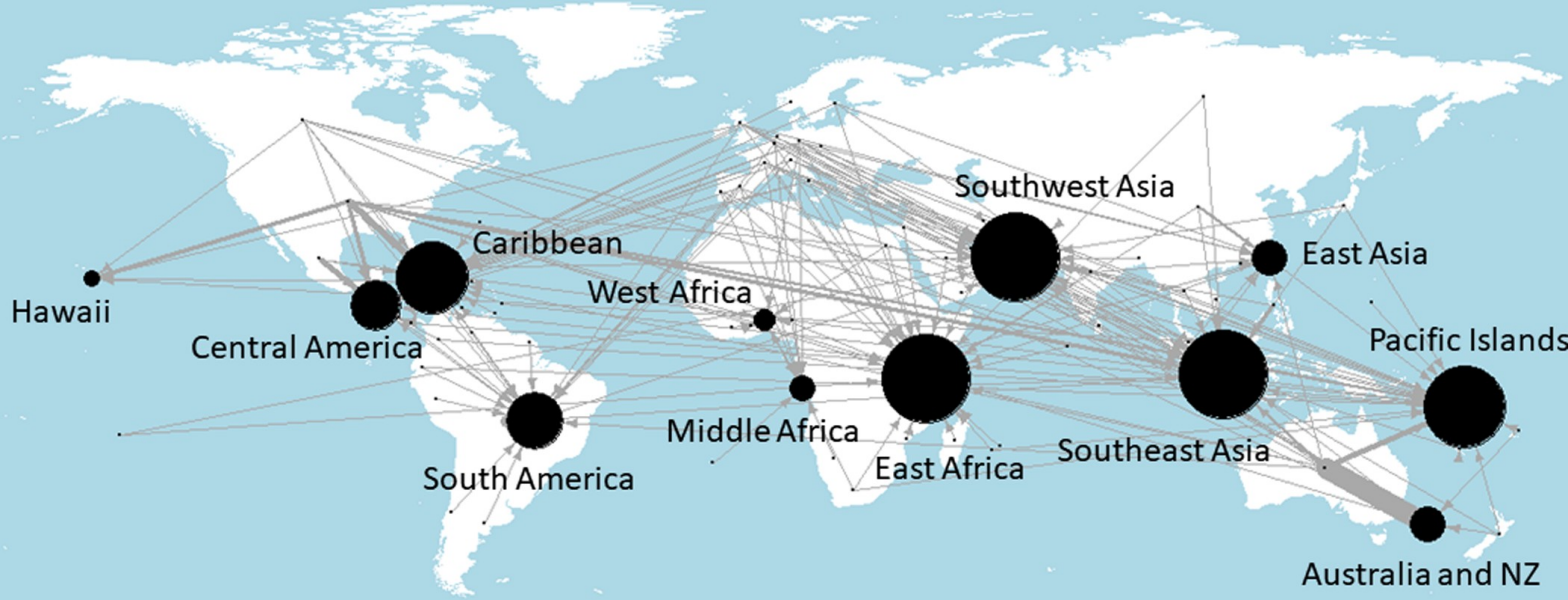
Directed network of the author country location with edge arrows going to the tropical region of research. Black circular nodes indicate research regions and the in-degree (i.e., the number of edges coming into the region).

The links between the origin (i.e., country affiliation) of the first author and the physical location (region) where the research was conducted or focused is shown in Fig 4. The map in Fig 4 shows two main pieces of information. First, first author countries are shown as small dots with directed arrow connections to the region where the research was conducted or focused on. The thickness of the arrow (i.e., edge connection) represents the number of publications. Second, the size of circle over each region represents the amount of publications in that region.

Southeast Asia, the Caribbean and the Pacific Islands, receive the most incoming research. This is followed by East Africa and South Asia. South America receives less incoming research, which also is reflected in Fig 3 by the high proportion of domestic collaborations in Brazil. Large portions of the USA research from only USA authors is conducted in Hawaii and the Caribbean, perhaps for spatial proximity reasons. Similarly, outgoing Australian research places substantial focus on the Pacific Islands. European outgoing research is more evenly scattered across regions.

### Citation rates and open access trends

**Fig 5:**
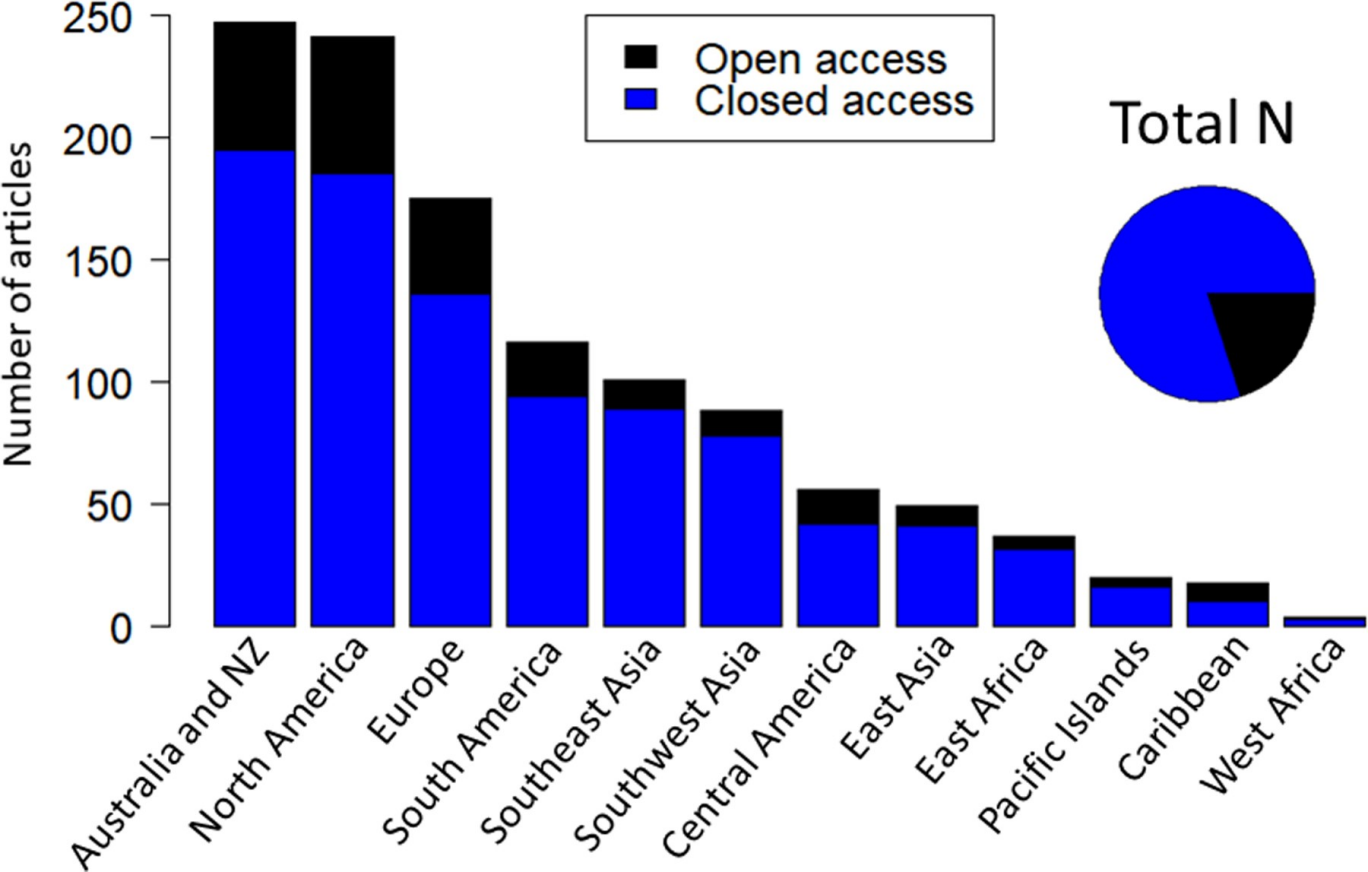
Open access publications versus closed access by region of the first author.

Citation rates and open access publication trends vary by region, where Western/ Northern regions lead most categories. Australia and NZ (New Zealand) lead all citation categories except percentage of papers with over 100 citations, where Europe and Southeast Asia have slightly more (Table 5). With a few exceptions, Australia and NZ, North America and Europe lead most citation categories. The sample size of the Pacific Islands is likely to small to represent and accurate percentage sample. For open access trends (Fig 5), the percentage of open access publications in regions with reliable sample sizes are in the following order: Central America (25%), North America (23%), Europe (22%), Australia and NZ (21%), South America (19%), East Asia (16%), East Africa (14%), Southeast Asia (12%), Southwest Asia (11%). The sample sizes of Pacific Islands, Caribbean and West Africa are likely too small to represent an accurate assessment. Approximately 20% of the total sample is open access.

**Table 5:** Publication citation data by region.

First author region	% missing citation data in sample	Avg. citations per pub. per year	Avg. citations per pub.	# pubs. with citation data	# and % pubs. with ≥50 citations	# and % pubs. with ≥100 citations
Australia and NZ	17.9	5.829	39.71	239	36 (15%)	11 (4.5%)
Caribbean	45.8	2.774	13.46	14	0	0
Central America	41.7	2.556	15.91	49	5 (10%)	1 (2%)
East Africa	28.0	2.335	9.88	36	1 (27%)	0
East Asia	20.0	2.878	20.36	44	3 (6.8%)	1 (2.2%)
Europe	21.3	3.663	25.75	166	22 (13%)	8 (4.8%)
North America	19.9	4.006	29.53	229	28 (12%)	10 (4.3%)
Pacific Islands	30.4	3.884	23.12	16	2 (12.5%)	1 (6%)
South America	28.2	2.863	13.64	102	5 (5%)	0
Southeast Asia	28.1	2.756	21.41	87	11 (12.6%)	6 (6.8%)
Southwest Asia	29.0	2.908	10.04	71	1 (1.5%)	0
West Africa	20.0	2.383	5.75	4	0	0

### Terminology use over space and time

**Fig 6:**
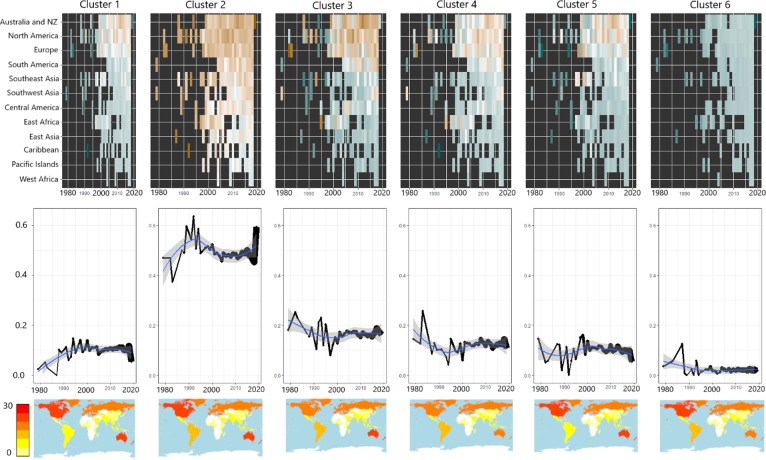
This figure has three parts. Top: Trajectories of the six terminology clusters identified from abstracts’ indicator words (1–6 Table 5 above) through space and time, as displayed through heat-maps of deviations from the occurrences of the cluster that would be expected if indicator words were uniformly distributed across clusters and regions in each year. Positive anomalies show increasing shades of brown, and negative anomalies turquoise (White = as expected if abstracts’ indicator words were even across clusters, space and time; Brown = more important; Turquoise = Less important; Black = No publication for that year and region). Mid: Time series of the contributions of each terminology to the total number of indicator words produced that year; increasing line widths represent the growing number of total publications (and hence: indicator words) through time. Bottom: Percentage contribution of each World Bank region to each terminology, averaged over time. White areas are countries with no first author publication.

Table 6 lists how 248 indicator words in article abstracts clustered together to form terminologies, while Fig 6 shows how these progressed through space (bottom), time (mid), and both (top). Australia, North America and Europe dominate each discourse, however taking different ranks (Fig 6, bottom). Generally, terminologies are not evenly distributed, see e.g., the high occurrence of cluster 4 in South America.

**Table 6:** Clustering of 248 indicator words into six terminology groups, based on their co-occurrence in 1328 abstracts.

Terminology clusters	Indicator words
Cluster 1	Fish, change, fishery, climate, understand, plan, process, research, information, scale, catch, future, global, state, knowledge, national, park, consider, examine, well, effort, government, action, focus, target, part, require, project, diverse, context, few
Cluster 2	Species, marine, use, management, community, high, ecosystem, habitat, water, island, tropical, sea, site, mangrove, increase, resource, conservation, ecological, impact, datum, local, provide, coast, system, may, sediment, level, population, effect, include, seagrass, model, development, large, low, suggest, zone, present, environment, activity, indicate, find, little, potential, there, approach, identify, some, good, human, need, biodiversity, natural, base, monitor, assess, significant, quality, protection, case, reduce, cause, affect, measure, occur, anthropogenic, threat, caribbean, importance, land, loss, when, strong, likely, recent, degradation, despite, function, adjacent, represent, available, decade, lead, throughout
Cluster 3	result, region, show, two, along, great, pattern, analysis, distribution, spatial, pacific, influence, year, current, factor, time, range, temperature, barrier, lagoon, south, role, variation, dynamic, size, difference, period, event, limit, variability, australia, world, small, individual, response, after, several, 2017, eastern, long-term, central, complex, surface, evidence
Cluster 4	ocean, diversity, abundance, value, structure, sample, cover, total, assemblage, survey, biomass, benthic, method, density, bay, map, composition, record, estimate, group, location, compare, relationship, depth, richness, indian, type, western, although, tool, variable, evaluate, index, biological, investigate, set, relative, reveal, control, demonstrate, 2018, term, overall
Cluster 5	condition, 5, economic, new, decline, support, strategy, major, under, social, tourism, mean, country, numb, paper, food, far, key, improve, here, pressure, four, benefit, status, health, challenge, main, live, critical, sustainable, contribute, policy, issue, exist, remain, aim, often
Cluster 6	Rate, nutrient, source, growth, concentration, determine, order, similar

Importance of the different clusters changed through time (Fig 6, mid), emerging (cluster 1), decreasing (cluster 4 and 6), or anticyclical fluctuations (clusters 2, 3 and 5). Cluster 2 was most prominent at all times, and 6 the least. The same patterns of dominance can be observed in the cluster heat-maps (Fig 6, top), with positive anomalies for Australia, North America and Europe as horizontal brown bars vs turquoise ones for most other regions; and cluster 2 having most positive, and 6 most negative anomalies. The simultaneous temporal and spatial resolution allows for detection of finer scale dynamics, where the emergence of a cluster can be seen in space (a region) and then observed how it moves (becomes more or less prevalent) across regions over time. In general, but most evidently in clusters 4 and 5, anomaly modes progressed from Europe in the years around 2000 to North America until the end of the decade, when the lead was taken over by Australia.

Throughout their initiation phase, some terminologies were more prominent in tropical regions than others. This accounts for the most abundant one, cluster 2, whereas cluster 1 was generally dominated through North America and Europe from the beginning. African, particularly East African first authors contributed visibly between 1995 and 2000, and disproportionally strong in clusters 2, 3 and 4.

While the terminological clusters may not represent what a more nuanced qualitative discourse analysis may reveal, it provides an initial empirical look into who may be driving discursive trends and change within the literature at a broad level. These findings enable the development of more nuanced, and more spatially and temporally specific hypotheses for further inquiry into the nexus between geopolitical relationships and science agendas.

## Discussion

The above scientometric analysis provides the quantitative empirical basis for observing how knowledge production trends are not evenly distributed across space, time or topic, supporting similar work on marine and coastal research globally [9,50]. The question of interest is not that trends should necessarily, or ever will be, evenly distributed, but rather if and why observed trends represent path dependent structures within the knowledge production system. If so, then the implications of this can be further hypothesized and discussed, which we suggest and elaborate on below. Science collaboration networks and publication trends play a large role in providing empirical data [51]. However, such findings require further theoretical analysis rooted in the social sciences to unpack the context under which scientific cooperation occurs (i.e., scientometrics need to be coupled with social theory to interpret their meaning in this context), and to develop research questions and hypotheses about why such trends are observed and what they mean in the wider context of scientific knowledge production. A transcultural and postcolonial Science and Technology Studies lens provides a useful social science theory to do so, providing established theoretical support behind the concept of path dependencies in sociology, political theory and economics [40,42,52]. Path dependency is useful for better understanding the meaning and context of our data and hypothesizing the historical and geopolitical realities that have shaped current agendas in tropical marine sciences, also demonstrating how other fields could be analyzed similarly.

In the following discussion, the detailed framework of path dependencies introduced in Table 1 is explored, drawing on the above empirical findings, existing literature and practical examples. However, upon elaboration of the various path dependencies below, their inseparability becomes clear, that they are by nature interconnected in ‘webs’ of dependencies that are created and reinforced by each other. As expressed by Matson et al., [53] in their 2016 book *Pursing Sustainability*, “academic communities by and large recognize today that there is no such thing as purely objective science. At a minimum, we choose our research areas subjectively, because of personal scientific interest in the topics, but we are also influenced in choosing them by public perceptions, funding opportunities, and our own beliefs and commitments,” (p. 140). Such subjectivities can be framed as path dependencies manifested within individuals, but the above empirical findings cannot be explained by or attributed to individual dependencies, but rather by the aggregation of their joint influence over space and time within broader structures of the science system, structures that can substantially shape what choices are available and made. Finally, as this research presents a case study of publication data analyzed with general scientometric methods, and interpreted through a broader path dependency lens, it may represent similar trends in other scientific fields. We conclude with more generalizable reflections on the global science system.

### Material science infrastructures

Marine Sciences, as an increasingly international system of largely English language based ocean-related knowledge production, irrespective of regional foci, are highly dependent on material infrastructures and technologies [54]. These range from large-scale and highly resource intensive infrastructures such as access to a research fleet (e.g., ships, submersibles), marine research stations, global ocean observatory systems, databases and models, to well-equipped laboratories and of course the transport infrastructures (e.g., air and sea ports) enabling access to the field research regions themselves [55–58], with increasing contestation over the justification for research activities and funds among disciplines [8]. Access to these material infrastructures substantially determines where and how data collection and analysis is conducted, with whose involvement as well as under which and whose agenda [59–61]. Patterns of marine science mirror an area-studies model despite arguments that marine space can provide an alternative lens to approach the spatial orientation of research [62]. The presented data illustrate that more than 50 percent of the lead authors of the assessed publication sample came from Australia, the U.S., Brazil or the U.K., while a negligible amount of contributing authors came from West Africa, the Caribbean, Southeast or Central Asia. Knowledge in the tropics is mostly not produced by the science systems within those countries. How scientists are connected to the people and places they research can be observed in publication networks, but these do not suggest why the network structures exist. We hypothesize that the subjects and objects of study are in part determined by infrastructure access, which is largely determined by funding in countries with available financing, and by networks of historical geopolitical relations in countries without their own funding.

In order to get an insight into the link between primary output of scientific publications and public and private sector investments into research and education, United Nations data can be observed due to the lack of available data for first-hand analysis on funding in scientific databases. For example, Gross Domestic Expenditure on Research and Development as a Percentage of GDP has been put forth by the UNESCO Institute of Statistics on the period between 1996 to 2017 [44]. Although these data are not specific to marine sciences, they show one of the most comprehensive and publically available assessments on science funding globally. It can be hypothesized that actors from well-funded science systems are more likely to be in the position to determine research agenda-setting processes, successfully conceptualize projects and act as first author on resulting publications. Following this, outcomes such as funding imbalances between countries and regions can be hypothesized as driving mechanisms that substantially co-shape scientific output production, and the observed authorship trends, in transregional networks. A systematic overlaying of the data on primary output production in the different countries from 1979–2018 with R&D investments in the field of marine and coastal sciences nevertheless unfortunately exceeded the scope of data analysis for this paper due to the lack of available data on funding in publication databases.

Those with substantially more funding opportunities will have more primary access to research infrastructures (i.e., research vessels, labs, transport), and are thus most likely to shape the agenda of the research and are proportionally more likely to be found under the primary authors of publications. Bouchet [57] states that “The Convention on Biological Diversity has highlighted the imbalance between the distribution of biodiversity and the distribution of knowledge on that biodiversity. Most known and unknown biodiversity is in tropical countries, most of them developing or emerging countries of the South, whereas most of the knowledge and resources on that biodiversity is in the developed countries of the North.” When looking at marine research stations in the tropics outside of the U.S. or Australia, those financing the stations seem to mainly determine the agenda of research and later publishing, not the science systems of the country they are located, [54]. At the same time, those countries with higher ocean science budgets and own access to tropical regions (e.g., the U.S., Australia) are shown above to focus their tropical marine scientific endeavors largely on their own tropical regions and ecosystems.

Limited access to material infrastructures is in some constellations compensated through respective transregional partnerships [63]. The data in this study can be interpreted as reflecting historical patterns of geopolitical network formation and the maintenance of past networks, supporting path dependency theory that this is typically less cost intensive than establishing new ones [40,42,64]. For example, when you think about doing your next scientific project, who will you do it with and where? It is likely that our previous partners, locations and case study regions are high on the list. Path dependency costs are real and can play out at all levels of science. The building of new networks in the interest of accessing needed material science infrastructures requires additional resources that are not required when working in established networks. As a result, established partnerships for the joint access to material infrastructures for science create strong dependencies on doing research in a location and a given network of partners [65,66]. A preference for maintaining our paths exists whether the reason was established in the best interest of science or not, or the relationship exists due to positive or negative historical geopolitical reasons or not.

### Immaterial science infrastructures

Besides access to material infrastructures, which is–as shown–often determined by immaterial infrastructures (e.g., science funding), additional immaterial science infrastructures act as powerfully guiding path dependencies on scientific knowledge production on tropical marine and coastal ecosystems. These include the scientific working languages in the respective systems, transregional collaboration networks [67] and the disciplinary, thematic or applied versus basic research focused organization of the science systems [8,63,65].

The interplay of several patterns in our analysis are illustrated and can be hypothesized as path dependencies. The transregional collaborative science outputs are substantially dominated by Western/ Northern countries whose science systems are equipped to set the agenda of the implemented research, as well as operate largely in English, the language of the publications assessed here empirically. Entirely Russian or Chinese language outputs are, for example, largely not archived in the popularized English language databases such as SCOPUS or Web of Science Core Collection, but it can be assumed that these non-English publications would have minimal international influence. In this paper we are interested primarily in international relationships. South-South collaborative publications hardly exist in the sample, which can be speculated by a lack of South-South international research projects in the field due to the lack of funding lines and donor institutions financially enabling this type of research, as well as the lack of joint traditions of doing collaborative research with scarcely developed transregional partnership networks between these countries. In line with this, most of the research is published outside of the countries in which the field research was conducted, and in fact outside the tropics, something that can be explained with the location of some of the major publishing houses for marine and sustainability-focused sciences outside of the tropics, as well as again the language of the assessed publications, namely English, being the international standard for the science systems.

As the ratio of domestic vs international co-authors is significantly correlated to the number of total co-authors, and domestic co-authorship ratios mirror increased total number of countries collaborated with, a few interpretations can be made. Countries with less total co-authors (also mirroring less total publications) only tend to publish with selected international co-authors when publishing in English, perhaps financed or working with projects financed by international partners (mostly North American, European or Australian). The lack of domestic co-authors in countries with relatively few papers suggest that either those countries do not publish, and thus do not influence the global science agenda in the field, perhaps due to lack of national funding as this data largely mirrors R&D country level investment per GDP, or these countries are only publishing/ and co-authoring domestically in a different language not sampled in this study. For many countries, this may be and is likely a combination of both. However, our observation that countries with few total collaborations do not tend to cooperate with each other but rather the few well financed Western/Northern countries suggests funding plays a substantial role in the web of dependencies guiding the structure of international networks.

Further, the terminological use to frame particular research questions and foci indicates a pattern of research agenda setting (indicated by terminology clusters) in Australia, North America and Europe and–with some time lag–that those agendas then spread globally. The content of the words within the clusters is less important, as we are interested in where they emerge and spread regionally, supporting other data showing asymmetric influences likely linked to funding and networks. Access to science funding can be hypothesized here as a key driver that facilitates the agenda and standard setting role of the Australian, North American and several European science systems [54]. Interestingly, Asian countries (including China and India) so far hardly feature as agenda setting entities in the field of tropical marine sciences, which is likely to be explained by the English language sample of publications assessed, because UNESCO-IOC (2017) assessed China as taking a frontrunner role in advancing numerous underwater technology development related fields.

Finally, disciplinary differences in publication behavior show that the natural sciences–by far–are the front runner in total publications across disciplines in the tropical marine sciences. This highlights the emphasis towards natural science disciplinary organization of the largely influential (in terms of publishing) national science systems, the institutional emphasis and predispositions for the natural sciences by funding agencies and its reciprocal approval by the tropical marine science communities themselves. The fact that natural sciences remain dominant within marine research in comparison to social sciences, despite the fact that the human dimension of the sea becomes more and more obvious, might be another path dependency. Future analyses could examine the data in terms of applied versus basic research produced, as well as a topic-oriented, interdisciplinary versus a disciplinary organization of research endeavors in the field.

### Asymmetry in global science systems

Scientifically assessing tropical coastal systems also means working in countries that have a colonial past, distant enclaves supporting larger empires with additional natural and human resources before becoming independent, and then shaped as ‘nation-states’. Political agendas were set, public discourses shaped, and thus ‘reality’ of everyday life, was substantially defined by geopolitical influences, such as the colonial powers and local elites working in the colonial apparatuses. Traces of colonially shaped science systems can until today be empirically observed in scientific knowledge production, agendas and natural resource management systems throughout the tropics [68–71].

Unequal power positions in negotiating social realities and imaginaries of the future—both aspects that lie at the heart of science and its role in society—are structurally embedded in many of the science systems in tropical nations with colonial legacies today, and are likely to prevail in negotiating scientific knowledge production and agendas globally. ‘Meeting on eye level’, whether between nationally bound, disciplinary, resource-focused or even gender-specific science and knowledge systems at large, is thus challenged by numerous structural path dependencies, regardless of the future directions desired for the science system going forward. The notions of ‘epistemic privilege’ and ‘epistemic oppression’ [72–74] speak of ‘epistemic inequalities’ that play out until today, where change and influence in the market place of knowledge and ideas is perhaps not an equal playing field for all involved. Although the data presented above do not show such detailed assumptions directly, trends in authorship networks and the patterns of Northern/ Western dominance in the field, coupled with UNESCO funding data, provide useful empirical data to match many of the claims made within Science and Technology Studies. Our cluster analysis suggests–at least discursively—that path dependencies exist within agenda setting processes.

In summary, our empirical analysis suggests, that access to material infrastructures (indicated by publication outputs) is determined by access to immaterial infrastructures, giving those science actors with access to viable funding a substantially more important role than those science actors with direct domestic tropical access itself (i.e., marine and coastal systems), but whom do not have access to the financial means necessary to finance the apparatuses or the capacity/power required for researching marine and coastal ecosystems in the tropics. With reference to de Solla Price [75], one can easily argue that indeed marine sciences are ‘big science’, but given the disproportionate influence of a small number of national science systems that have the required funding for this big science, this translates into agenda setting being done by few (as indicated by UNESCO-IOC 2017).

Although they are a ‘big science’, marine sciences are not a ‘global science’ in this regard. Existing, partly since (pre-)colonial times, transregional partnership networks act as institutions facilitating access to the necessary but hard to break webs of dependencies–to the field, to science infrastructures, to science funds, and science agendas. Although such dependencies tend to be reinforced reciprocally and in part knowingly on both sides, for example with money and theoretical knowledge in exchange for access/permits and local knowledge (e.g., language/ guides), the beneficiaries of big science are clear in short term (see ongoing politics of science collaborations [76–78]). Established partnerships, whether characterized by equal partnership or not, exist as path dependencies that substantially guide future research collaboration shaping the type of scientific knowledge that is being produced, largely because patterns within these networks are difficult to break, and often beyond the scope of influence for individual researches to change. By doing so, the myriad forms of networked dependencies within our science institutions repeatedly affirm mutually imbalanced relationships between agenda setting science systems and agenda co-implementing science systems providing access to the field and tropical world. From this perspective we can see that the production of science that intends to meet at eye level among internationally collaboration institutions in the future is a collective action problem, a clash between individuals largely locked within path dependent patterns of international collaboration, while the interest of the broader science system is to incentivize the deviation away from the negative aspects of those patterns.

Marine sciences are a highly international field [9,54]. This article has aimed to unpack what exactly this means in practice, and to critically identify defining characteristics co-shaping marine scientific knowledge production on and in the tropics. The high degree of internationality within the field also means that tropical marine sciences can indeed play a crucial role in changing old and potentially problematic (e.g., disempowering) patterns of interaction between ‘North/West’ and ‘South/East’ and the transregional power imbalances that continue hampering transregional science cooperation ‘on eye level’. How our data can nevertheless be interpreted is that the need to consciously reflect on the current role of tropical marine sciences in, either, further affirming existing divides between the respective science systems involved, or, substantially contributing to overcoming this divide by fostering actual joint collaboration. Perhaps, starting with jointly formulating research agendas, jointly looking for funding, which should go beyond the nation state, jointly conducting the research and finally taking equal turns in taking the lead in publishing the results [79]. One might ask, when does the ‘epistemic privilege’ of the well-funded marine science systems of North America, Australia and Europe result in negative or disempowering patterns [72–74] for the research partners in the tropics–who do not define the global research agenda dominated by the English language, but largely arrange the practicalities for accessing the field (e.g., research licenses/ permits, the provision of research assistants and translators), only later acting as local correctives in writing processes (often by national mandates for co-authorship by the partner country, or for maintaining geopolitical relations or field access for future projects), but rarely leading them. Many benefits are of course gained for the cooperating science systems within these partnerships, but reflection is also needed on the potential downsides.

It is not the intention of this article to suggest that epistemic oppression can be directly observed in the field of tropical marine sciences, we don’t have such data and it can only be interpreted with social theories over scientometrically analyzed data, and scientometric analysis will likely never show such data without attempts to connect the interpretation of what those data mean in practice through social theory, such as Science and Technology Studies. Rather it is the intention of the article to motivate critical reflection on a broader notion of sustainability that includes recognizing the role of systematic institutional incentives and drivers at the global and international level that shape science as a knowledge production and geopolitical nexus. Broader notions of sustainability are not only about recognizing the links between social and environmental change, and the normative preferences for addressing them, but also the very role of the science systems themselves that shape how that knowledge is produced and used. Thus, this article aims to (a) take indications in the data seriously, that epistemic inequality, and thus a substantial divide and unequal role division and sharing within transregional research collaborations is interpretable through global level scientometric analysis when layered with theories of path dependency—linking the English agenda setting science systems and their researchers as well as the agenda-following science systems and their researchers; (b) understand the interconnected webs of path dependencies, even if largely interpreted theoretically, that are responsible for continuously reaffirming, and thus deepening, asymmetric reinforcement of global knowledge production; and (c) enable empirically-informed decision-making on the level of science policy-makers and scientists themselves when designing funding and program lines, as well as proposals.

The international production of tropical marine scientific knowledge–disproportionately influenced by the Global North, whom have little access to the physical tropical world—assure that the tropics and their inhabitants remain research objects, rather than becoming research subjects and thus agents of change themselves. The sustainable use and management of tropical marine ecosystems, for which tropical marine sciences are to provide the scientific basis, nevertheless can only be achieved together with these agents of change from within, from the marine sciences in the tropics themselves. Addressing the above identified path dependencies that shape the asymmetric relationships within transregional collaborations until today, whether or not they are perceived to be normatively disempowering or inequitable, are not a point of ethics or human values. They are simply empirically observable and interpretable. If indeed a sustainable use and management of tropical marine ecosystems shall be achieved, it will be reliant on identifying and critically reflecting on the role of science as an institutional apparatus with systematic and path dependent patterns that can be observed globally, and likely within numerous other fields beyond tropical marine sciences.

## Supporting information

S1 Data (CSV)

S2 Data (CSV)

S3 Data (CSV)

S4 Data (CSV)

S5 Data (CSV)

S1 Fig Changes in pearson’s correlation between the underlying Ochiai dissimilarity matrix and binary distance matrices computed from the dendogram cut at k, and at k-1 groups. Increasing the number of clusters from one to two produced the largest increase in correlation (red dot). We used the second, more informative maximum of six groups (green dot) for the final partitioning.
(DOCX)

S2 Fig Dendogram of 248 indicator words from 1328 abstracts clustered into 6 terminology groups. (DOCX)

S3 Fig Check of assumptions in the linear regression model. (DOCX)

S4 Fig Scatterplots of collaboration data in Fig 2 with univariate linear model fit line. (DOCX)

S1 Table Total number of first author and total authors per country. (DOCX)

S2 Table The disciplinary focus of articles grouped into categories. Categories include the following disciplinary compositions: ‘Natural’ includes ecology, biology, chemistry, geology; ‘Social’ includes political, sociology, anthropology, economics, history; ‘Other’ includes geography, sustainability science other. Data only until 2014 (n = 753).
(DOCX)

S3 Table Lemmatization and removal of non-informative nouns, and words, from the most common words of abstracts on marine tropical science, as performed for the cluster analysis to identify assemblages of words that could represent different terminologies (c.f., Methods: *Terminological cluster analysis*). Words that were not a noun (“True” in the third column) were removed from the data set, unless they were considered informative (“True” in the fourth column).
(DOCX)

S4 Table Step-by-step systematic review procedure. (DOCX)

S5 Table Node (country) centrality metrics for the undirected authorship network. (DOCX)

S6 Table Network metric definitions. (DOCX)

S7 Table Unique collaborations with other countries. (DOCX)

S8 Table Linear regression model. (DOCX)

## References

[1] Karaulova M, Gök A, Shackleton O, Shapira P. Science system path-dependencies and their influences: nanotechnology research in Russia. Scientometrics. 2016;107(2):645–70. 27122645 10.1007/s11192-016-1916-3 PMC4833821

[2] Aistleitner M, Kapeller J, Steinerberger S, Steinerberger S. The Power of Scientometrics and the Development of Economics. J Econ Issues. 2018;52(3):816–34.

[3] Heimeriks G, Boschma R. The path- and place-dependent nature of scientific knowledge production in biotech 1986–2008. 2013;1–26.

[4] Rafols I, Porter AL, Leydesdorff L. Science overlay maps: a new tool for research policy and library management. J Am Soc Inf Sci Technol. 2010;61(9):1871–87.

[5] Bruhn M, Gallego FA. GOOD, BAD, AND UGLY COLONIAL ACTIVITIES: DO THEY MATTER FOR ECONOMIC DEVELOPMENT? Rev Econ Stat. 2012;94(2):433–61.

[6] UNESCO-IOC. Global Ocean Science Report: The Current Status of Ocean Science Around the World. Paris: UNESCO; 2017. 271 p.

[7] Glaser M, Christie P, Diele K, Dsikowitzky L, Ferse S, Nordhaus I, et al Measuring and understanding sustainability-enhancing processes in tropical coastal and marine social–ecological systems. Curr Opin Environ Sustain [Internet]. 2012 7 [cited 2014 Jul 16];4(3):300–8. Available from: http://linkinghub.elsevier.com/retrieve/pii/S1877343512000590

[8] Markus T, Hillebrand H, Hornidge A-K, Krause G, Schlüter A. Disciplinary diversity in marine sciences: the urgent case for an integration of research. ICES J Mar Sci [Internet]. 2017;75(2):502–509. Available from: https://academic.oup.com/icesjms/article-lookup/doi/10.1093/icesjms/fsw213

[9] Partelow S, Schlüter A, von Wehrden H, Jänig M, Senff P. A Sustainability Agenda for Tropical Marine Science. Conserv Lett [Internet]. 2018;11(1). Available from: http://doi.wiley.com/10.1111/conl.12351

[10] Helmreich S. Alien Ocean: Anthropological Voyages in Microbial Seas. Berkeley: University of California Press; 2009.

[11] Hamblin JD. Oceanographers and the Cold War: Disciples of Marine Science. Seattle: University of Washington Press; 2005.

[12] Heidbrink I. Deutschlands einzige Kolonie ist das Meer: Die deutsche Hochseefischerei und die Fischereikonflikte des 20. Hamburg: Jahrhunderts; 2004.

[13] Gillis JR. The human shore: Seacoasts in history. University of Chicago Press; 2012.

[14] Blum H. The Prospect of Oceanic Studies. Pmla. 2010;125(3):670–7.

[15] De Bont R. Between the laboratory and the deep blue sea: Space issues in the marine stations of Naples and Wimereux. Soc Stud Sci. 2009;39(2):199–227. 10.1177/0306312708097325 19831221

[16] Mancke E. Early Modern Expansion and the Politicization of Oceanic Space. Geogr Rev. 1998;89(2):225–36.

[17] Lenz W, Deacon M. Ocean Sciences: Their History and Relation to Man In: Ergänzungsheft Deutsche Hydrographische Zeitschrift. Hamburg: Reihe B/22; 1990.

[18] Hornidge A-K. A Research Vessel. Heterotopia, Boundary Place and Pluriverse of Epistemes In: Poferl A, Pfadenhauer M, editors. Wissensrelationen: Beiträge und Debatten zum 2 Sektionskongress der Wissenssoziologie. BELTZ JUVENTA; 2018.

[19] Hubbard J. A Science on the Scales: the Rise of Canadian Atlantic Fisheries Biology 1898–1939. Toronto: Toronto University Press; 2006.

[20] Helmreich S. A Tale of Three Seas: From Fishing through Aquaculture to Marine Biotechnology in the Life History Narrative of a Mariue Biologist. Marit Stud. 2003;2(2):73–94.

[21] Adams J. The rise of research networks. Nature. 2012;490:335–6. 10.1038/490335a 23075965

[22] Gattuso J, Dawson NA, Duarte CM, Middelburg JJ. Patterns of publication effort in coastal biogeochemistry: a bibliometric survey (1971 to 2003). Mar Ecol Prog Ser. 2005;294:9–22.

[23] Elango B, Rajendran P. Authorship Trends and Collaboration Pattern in the Marine Sciences Literature: A Scientometric Study. Int J Inf Dissem Technol. 2012;2(3):166–9.

[24] Shen S, Cheng C, Yang J, Yang S. Visualized analysis of developing trends and hot topics in natural disaster research. PLoS One [Internet]. 2018;13(1):1–15. Available from: 10.1371/journal.pone.0191250 PMC5774750 29351350

[25] Aznar-Sánchez JA, Piquer-Rodríguez M, Velasco-Muñoz JF, Manzano-Agugliaro F. Worldwide research trends on sustainable land use in agriculture. Land use policy. 2019;87(February).

[26] Leydesdorff L, Wagner CS. International collaboration in science and the formation of a core group. J Informetr. 2008;2(4):317–25.

[27] Cash-Gibson L, Rojas-Gualdrón DF, Pericàs JM, Benach J. Inequalities in global health inequalities research: A 50-year bibliometric analysis (1966–2015). PLoS One. 2018;13(1):1–22. 10.1371/journal.pone.0191901 PMC5792017 29385197

[28] KIM K-W. Measuring international research collaboration of peripheral countries: Taking the context. Scientometrics. 2006;66(2):231–40.

[29] Glänzel W, Zhang L. Scientometric research assessment in the developing world: A tribute to Michael J. Moravcsik from the perspective of the twenty-first century. Scientometrics. 2018;115(3):1517–32.

[30] Syed S, ní Aodha L, Scougal C, Spruit M. Mapping the global network of fisheries science collaboration. Fish Fish. 2019;(October 2018):1–27.

[31] Barboza LGA, Gimenez BCG. Microplastics in the marine environment: Current trends and future perspectives. Mar Pollut Bull. 2015;97:5–12. 10.1016/j.marpolbul.2015.06.008 26072046

[32] Mazaris AD, Gkazinou C, Almpanidou V, Balazs G. The sociology of sea turtle research: evidence on a global expansion of co-authorship networks. Biodivers Conserv [Internet]. 2018;27(6):1503–16. Available from: 10.1007/s10531-018-1506-1

[33] Fourqurean JW, Duarte CM, Kershaw MD, Threlkeld ST. Estuaries and Coasts as an Outlet for Research in Coastal Ecosystems: A Bibliometric Study. Estuaries and Coasts. 2008;31:469–76.

[34] Kim J, Lee S, Shim W, Kang J. A mapping of marine biodiversity research trends and collaboration in the east Asia region from 1996–2015. Sustain. 2016;8(10).

[35] Evers HD, Hornidge AK. Knowledge hubs along the Straits of Malacca. Asia Eur J. 2007;5(3):417–33.

[36] Schubert T, Sooryamoorthy R. Can the centre-periphery model explain patterns of international scientific collaboration among threshold and industrialised countries? The case of South Africa and Germany. Scientometrics. 2010;83(1):181–203.

[37] Zdravkovic M, Chiwona-Karltun L, Zink E. Experiences and perceptions of South–South and North–South scientific collaboration of mathematicians, physicists and chemists from five southern African universities. Scientometrics. 2016;108(2):717–43.

[38] Aksnes DW, Browman HI. An overview of global research effort in fisheries science. ICES J ofMarine Sci. 2016;73(4):1004–11.

[39] Marrero ME, Payne DL, Breidahl H. The Case for Collaboration to Foster Global Ocean Literacy. Front Mar Sci. 2019;6(February 2007):1–2.

[40] Mahoney J. Path dependence in historical sociology. Theory Soc. 2000;29(4):507–48.

[41] North DC. Institutions, Institutional Change, and Economic Performance. Cambridge Univ Press. 1990;5(1):1–153.

[42] Pierson P. Increasing Returns, Path Dependence, and the Study of Politics. Am Polit Sci Rev. 2000;94(2):251–67.

[43] van Assche K, Beunen R, Duineveld M. Evolutionary Governance Theory [Internet]. 2014. Available from: http://link.springer.com/10.1007/978-3-319-00984-1

[44] UNESCO Institue for Statistics. Global trends in R&D expenditure [Internet]. UNESCO UIS UNESCO eAtlas of Research and Experimental Development. 2018 [cited 2019 Nov 5]. Available from: https://www.tellmaps.com/uis/rd/#!/tellmap/-680879682

[45] UNESCO. UNESCO Global Science Report: Towards 2030. Paris; 2015.

[46] Csardi G, Nepusz T. igraph: Network analysis and visualization. R package; 2015.

[47] MaxQDA. MaxQDA. Berlin, Germany: VERBI Software; 2016.

[48] R Core Team. R: A language and environment for statistical computing [Internet]. Vienna, Austria; 2018. Available from: http://www.r-project.org/

[49] Borcard D, Gillet F, Legendre P. Spatial analysis of ecological data In: Numerical ecology with R. New York: Springer; 2011 p. 227–92.

[50] Liquete C, Piroddi C, Drakou EG, Gurney L, Katsanevakis S, Charef A, et al Current Status and Future Prospects for the Assessment of Marine and Coastal Ecosystem Services: A Systematic Review. PLoS One. 2013;8(7). 10.1371/journal.pone.0067737 PMC3701056 23844080

[51] Shrum W., Genuth J., Chompalov I., Carlson W. B., & Bijker WE. Structures of scientific collaboration. MIT Press; 2007.

[52] David PA. Path dependence: A foundational concept for historical social science. Cliometrica. 2007;1(2):91–114.

[53] Matson P, Clark WC, Andersson K. Pursuing Sustainability: A Guide to the Science and Practice. Princeton University Press; 2016 231 p.

[54] Isensee, K., Horn, L. and Schaaper M. The funding for ocean science. In: Global Ocean Science Report—The current status of ocean science around the world. Paris: IOC- UNESCO; 2017. p. 80–97.

[55] Martino J. Science Funding [Internet]. 1st ed. New York: Routledge; 1992. Available from: 10.4324/9781351294768

[56] JUDA L. Changing National Approaches to Ocean Governance: The United States, Canada, and Australia. Ocean Dev Int Law. 2003;34(2):161–87.

[57] Bouchet P. The magnitude of marine biodiversity In: Duarte CM, editor. The Exploration of Marine Biodiversity: Scientific and Technological Challenges. Fundacion BBVA; 2006.

[58] Mueller M, Wallace R. Enabling science and technology for marine renewable energy. Energy Policy. 2008;36:4376–82.

[59] Maclean M, Anderson J, Martin BR. Identifying research priorities in public sector funding agencies: mapping science outputs on to user needs. Technol Anal Strateg Manag. 1998;10(2):139–55.

[60] Jacquet J, Pauly D. Funding Priorities: Big Barriers to Small-Scale Fisheries. Conserv Biol. 2008;22(4):832–5. 10.1111/j.1523-1739.2008.00978.x 18637910

[61] Clifton J. Science, funding and participation: key issues for marine protected area networks and the Coral Triangle Initiative. Environ Conserv. 2009;36(2):91–6.

[62] Lewis MW, Wigen K. A MARITIME RESPONSE TO THE CRISIS IN AREA STUDIES. Geogr J. 1999;89(2):161–8.

[63] Wescott G. Partnerships for capacity building: community, governments and universities working together. Ocean Coast Manag. 2002;45:549–71.

[64] Collier RB, Collier D. Shaping the Political Arena: Critical junctures, the labor movement, and regime dynamics in Latin America. Notre Dame: University of Notre Dame Press; 1991.

[65] Holm P, Evan M, Cloetingh S, Agnoletti M, Moldan B, Lang DJ, et al Collaboration between the natural, social and human sciences in Global Change Research. Environ Sci Policy. 2013;28.

[66] Hind EJ, Alexander SM, Green SJ, Kritzer JP, Sweet MJ, Johnson AE, et al Fostering effective international collaboration for marine science in small island states. Front Mar Sci. 2015;2(October):1–7.

[67] Hennemann S, Rybski D, Liefner I. The Myth of Global Science Collaboration. J Informetr. 2012;6(2):217–25.

[68] Agrawal A. Environmentality: technologies of government and the making of subjects. Duke University Press; 2005.

[69] Cashman K. Systems of knowledge as systems of domination: The limitations of established meaning. Agric Human Values. 1991;8(1–2):49–58.

[70] Conrad S, Randeria S, Römhild R. Jenseits des Eurozentrismus: Postkoloniale Perspektiven in den Geschichts- und Kulturwissenschaften. Frankfurt and New York: Campus Verlag; 2013.

[71] Hornidge A-K, Shtaltovna A, Schetter C. Agricultural Knowledge and Knowledge Systems in Post-Soviet Societies. Hornidge A-K, Shtaltovna A, Schetter C, editors. Bern: Bern: Peter Lang; 2016.

[72] Dotson K. Conceptualizing Epistemic Oppression. Soc Epistemol. 2014;28(2):115–38.

[73] Fricker M. Epistemic Injustice: Power and the Ethics of Knowing. Oxford: Oxford University Press; 2007.

[74] Fricker M. Epistemic Oppression and Epistemic Privilege. Can J Philos [Internet]. 1999;25:191–210. Available from: http://www.tandfonline.com/doi/full/10.1080/00455091.1999.10716836

[75] Price DJ de S. Little science, big science … and beyond. New York: Columbia University Press; 1986 5 p.

[76] Rochmyaningsih D. Indonesia plans strict foreign research laws. Nature. 2018;557(7706):476 10.1038/d41586-018-05001-7 29789750

[77] Mervis J, Cho A. New DOE policies would block many foreign research collaborations. Science [Internet]. 2019; Available from: https://www.sciencemag.org/news/2019/02/new-doe-policies-would-block-many-foreign-research-collaborations

[78] Science without walls is good for all. Nature [Internet]. 2017; Available from: https://www.nature.com/news/science-without-walls-is-good-for-all-1.22742 10.1038/550007b 28980659

[79] Sarna-Wojcicki D, Perret M, Eitzel M V, Fortmann L. Where Are the Missing Coauthors? Authorship Practices in Participatory Research. Rural Sociol. 2017;00(00).

