# Supplementary material for: Tropical marine sciences: Knowledge production in a web of path dependencies
Source: PLoS One. 2020 Feb 6;15(2):e0228613. doi: 10.1371/journal.pone.0228613 (PMC7004553; doi:10.1371/journal.pone.0228613)
Supplement: S1 Fig — Increasing the number of clusters from one to two produced the largest increase in correlation (red dot). We used the second, more informative maximum of six groups (green dot) for the final partitioning. (DOCX) [file pone.0228613.s006.docx]

**
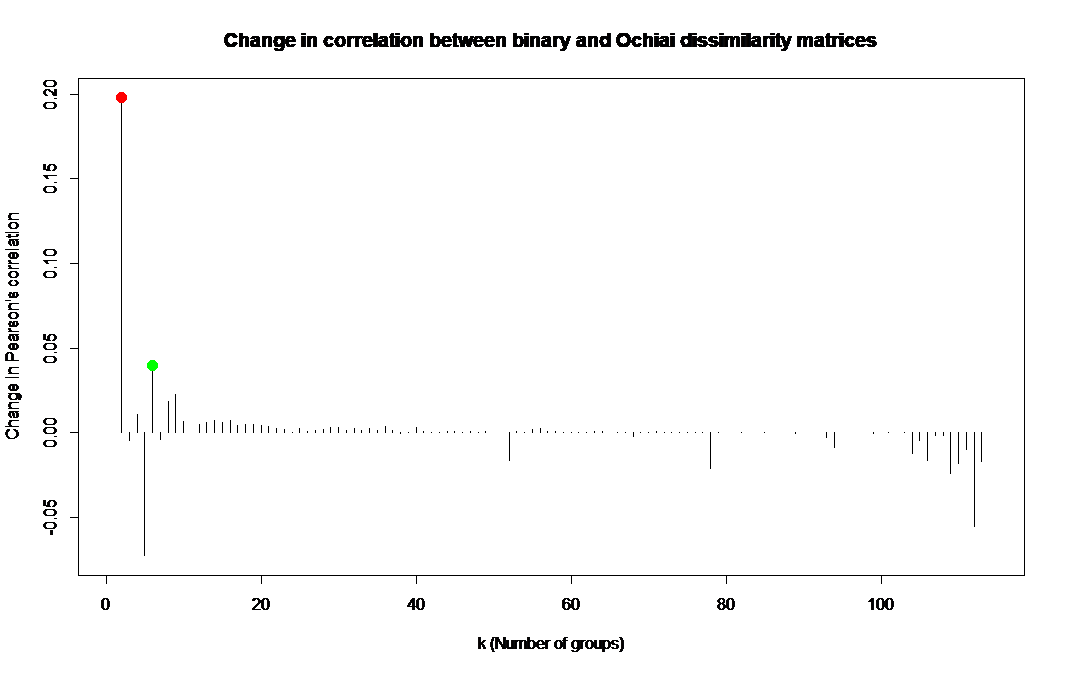
**

**Figure S1**. Changes in Pearson’s correlation between the underlying Ochiai dissimilarity matrix and binary distance matrices computed from the dendogram cut at k, and at k-1 groups. Increasing the number of clusters from one to two produced the largest increase in correlation (red dot). We used the second, more informative maximum of six groups (green dot) for the final partitioning.
