# Supplementary figures and images for: Tropical marine sciences: Knowledge production in a web of path dependencies
Source: PLoS One. 2020 Feb 6;15(2):e0228613. doi: 10.1371/journal.pone.0228613 (PMC7004553; doi:10.1371/journal.pone.0228613)

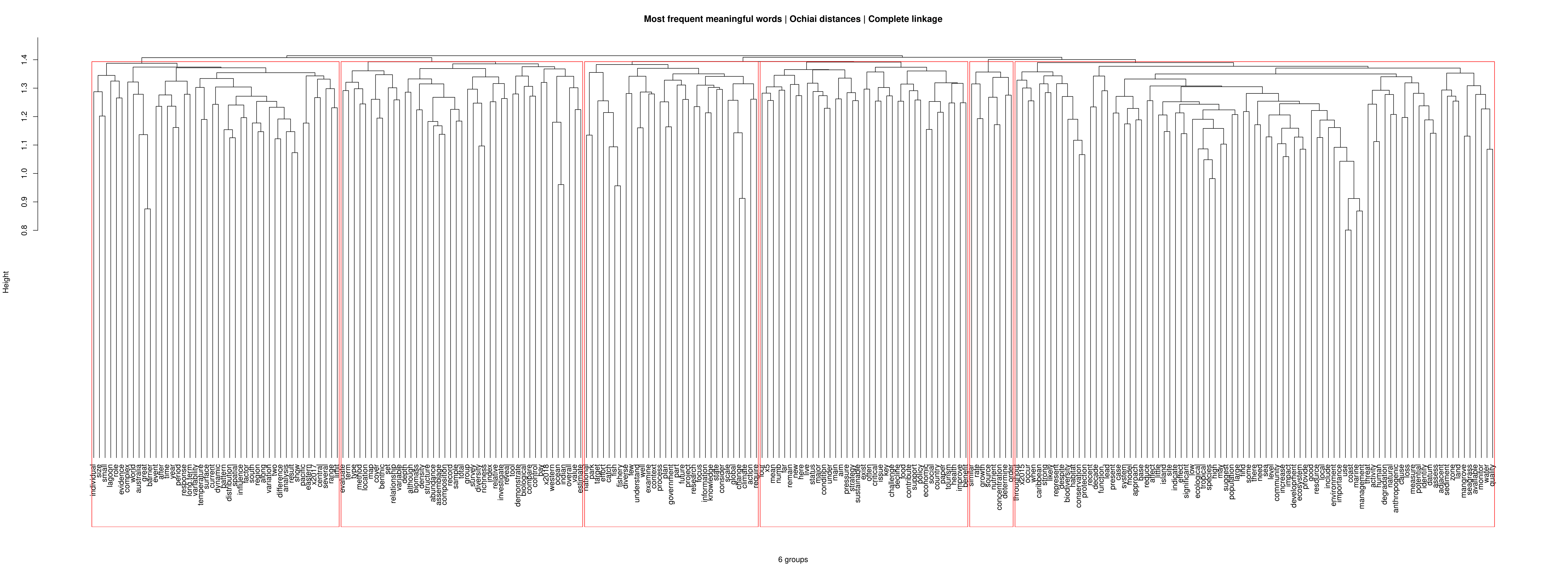


**Figure S2**. Dendogram of 248 indicator words from 1328 abstracts clustered into 6 terminology groups.

Supplement: S2 Fig — (DOCX) [file pone.0228613.s007.docx]

**
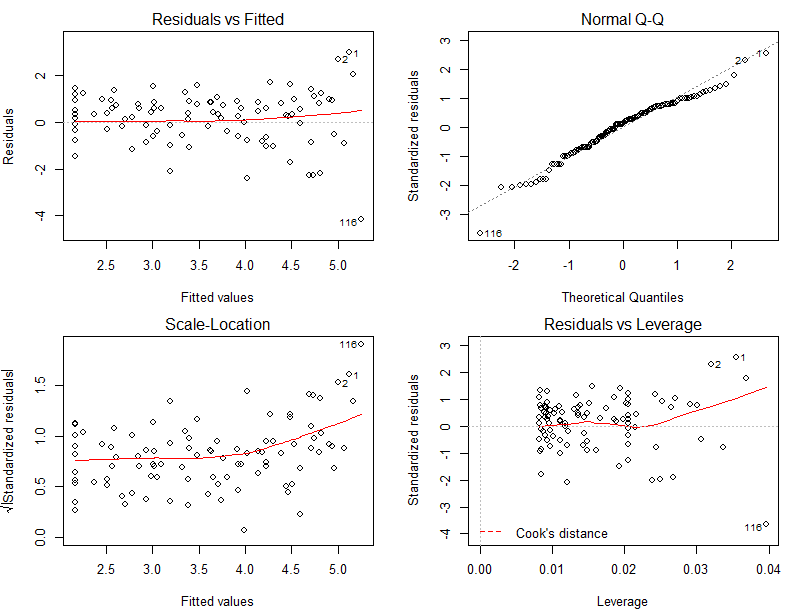
**

**Figure S3.** Check of assumptions in the linear regression model.

Supplement: S3 Fig — (DOCX) [file pone.0228613.s008.docx]

**Figure S4**. Scatterplots of collaboration data in Figure 2 with univariate linear model fit line.


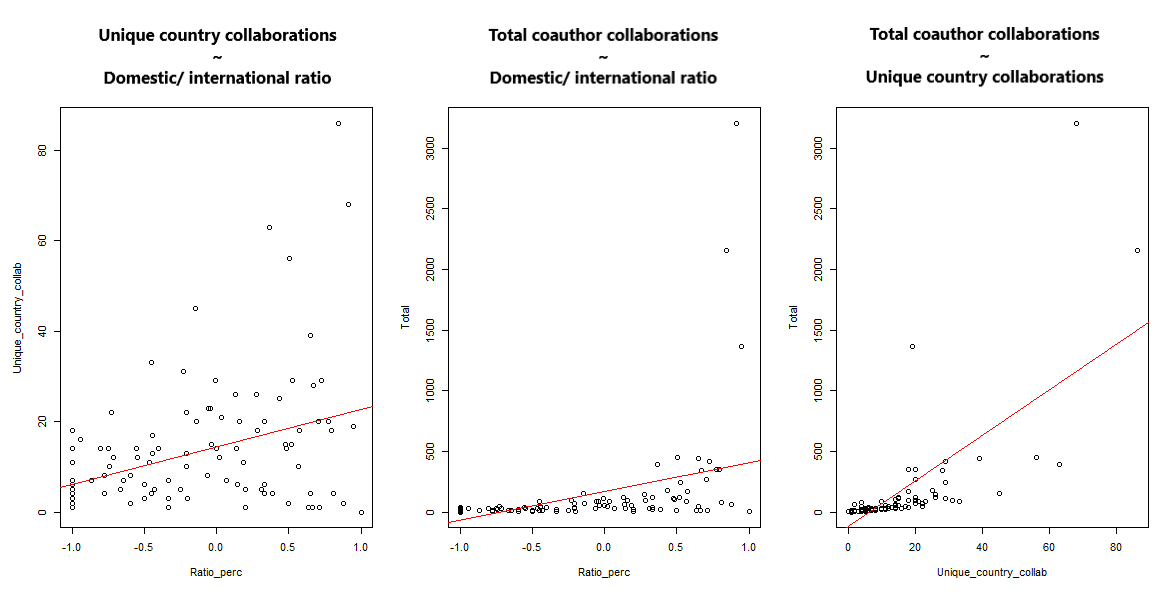

Supplement: S4 Fig — (DOCX) [file pone.0228613.s009.docx]
