# Supplementary material for: Tropical marine sciences: Knowledge production in a web of path dependencies
Source: PLoS One. 2020 Feb 6;15(2):e0228613. doi: 10.1371/journal.pone.0228613 (PMC7004553; doi:10.1371/journal.pone.0228613)
Supplement: S1 Table — (DOCX) [file pone.0228613.s010.docx]

**Supplementary Information (SI Appendix)**

**Table S1.** Total number of first author and total authors per country.

| **Country** | **Percentage lead author** | **Total N lead author** | **Percentage all authors** | **Total N all authors** |
| --- | --- | --- | --- | --- |
| Australia | 20.26 | 269 | 20.03 | 1210 |
| USA | 18.07 | 240 | 17.48 | 1056 |
| Brazil | 8.21 | 109 | 8.16 | 493 |
| UK | 4.89 | 65 | 4.55 | 275 |
| India | 3.99 | 53 | 3.18 | 192 |
| Mexico | 3.99 | 53 | 3.69 | 223 |
| France | 3.09 | 41 | 3.66 | 221 |
| Philippines | 2.71 | 36 | 2.80 | 169 |
| Germany | 2.64 | 35 | 2.65 | 160 |
| Canada | 2.48 | 33 | 1.94 | 117 |
| China | 2.18 | 29 | 2.33 | 141 |
| Malaysia | 1.81 | 24 | 1.79 | 108 |
| Kenya | 1.20 | 16 | 1.22 | 74 |
| Costa Rica | 1.13 | 15 | 1.09 | 66 |
| Indonesia | 1.13 | 15 | 1.69 | 102 |
| Singapore | 1.13 | 15 | 1.41 | 85 |
| Sweden | 1.13 | 15 | 1.11 | 67 |
| Thailand | 0.98 | 13 | 0.84 | 51 |
| New Caledonia | 0.83 | 11 | 0.89 | 54 |
| Tanzania | 0.83 | 11 | 0.71 | 43 |
| Colombia | 0.75 | 10 | 0.65 | 39 |
| Japan | 0.75 | 10 | 0.74 | 45 |
| Taiwan | 0.75 | 10 | 0.86 | 52 |
| Netherlands | 0.68 | 9 | 0.81 | 49 |
| Spain | 0.68 | 9 | 1.08 | 65 |
| Egypt | 0.60 | 8 | 0.30 | 18 |
| Italy | 0.60 | 8 | 0.91 | 55 |
| Panama | 0.60 | 8 | 0.51 | 31 |
| Saudi Arabia | 0.60 | 8 | 0.55 | 33 |
| Vietnam | 0.60 | 8 | 0.48 | 29 |
| Bangladesh | 0.53 | 7 | 0.50 | 30 |
| South Africa | 0.53 | 7 | 0.60 | 36 |
| Cuba | 0.45 | 6 | 0.55 | 33 |
| Ecuador | 0.45 | 6 | 0.33 | 20 |
| New Zealand | 0.38 | 5 | 0.38 | 23 |
| Puerto Rico | 0.38 | 5 | 0.22 | 13 |
| Switzerland | 0.38 | 5 | 0.28 | 17 |
| UAE | 0.38 | 5 | 0.25 | 15 |
| Belgium | 0.38 | 5 | 0.68 | 41 |
| Brunei | 0.32 | 4 | 0.10 | 6 |
| Trinidad and Tobago | 0.31 | 4 | 0.15 | 9 |
| Fiji | 0.30 | 4 | 0.26 | 16 |
| Iran | 0.30 | 4 | 0.18 | 11 |
| Jamaica | 0.30 | 4 | 0.22 | 13 |
| Portugal | 0.30 | 4 | 0.28 | 17 |
| Russia | 0.30 | 4 | 0.28 | 17 |
| Sri Lanka | 0.30 | 4 | 0.23 | 14 |
| Chile | 0.23 | 3 | 0.08 | 5 |
| Hong Kong | 0.23 | 3 | 0.31 | 19 |
| Israel | 0.23 | 3 | 0.20 | 12 |
| Oman | 0.23 | 3 | 0.22 | 13 |
| Venezuela | 0.23 | 3 | 0.23 | 14 |
| Bahamas | 0.15 | 2 | 0.17 | 10 |
| Barbados | 0.15 | 2 | 0.22 | 13 |
| Czech Republic | 0.15 | 2 | 0.03 | 2 |
| French Polynesia | 0.15 | 2 | 0.28 | 17 |
| Guam | 0.15 | 2 | 0.13 | 8 |
| La Reunion | 0.15 | 2 | 0.25 | 15 |
| Mauritius | 0.15 | 2 | 0.22 | 13 |
| Netherlands Antilles | 0.15 | 2 | 0.15 | 9 |
| Norway | 0.15 | 2 | 0.13 | 8 |
| Pakistan | 0.15 | 2 | 0.08 | 5 |
| Peru | 0.15 | 2 | 0.33 | 20 |
| Solomon Islands | 0.15 | 2 | 0.22 | 13 |
| Argentina | 0.08 | 1 | 0.07 | 4 |
| Bahrain | 0.08 | 1 | 0.12 | 7 |
| Belize | 0.08 | 1 | 0.07 | 4 |
| Benin | 0.08 | 1 | 0.07 | 4 |
| Bermuda | 0.08 | 1 | 0.05 | 3 |
| El Salvador | 0.08 | 1 | 0.03 | 2 |
| French Guiana | 0.08 | 1 | 0.02 | 1 |
| Ghana | 0.08 | 1 | 0.07 | 4 |
| Ivory Coast | 0.08 | 1 | 0.08 | 5 |
| Madagascar | 0.08 | 1 | 0.22 | 13 |
| Maldives | 0.08 | 1 | 0.08 | 5 |
| Mozambique | 0.08 | 1 | 0.08 | 5 |
| Namibia | 0.08 | 1 | 0.05 | 3 |
| Nicaragua | 0.08 | 1 | 0.15 | 9 |
| Nigeria | 0.08 | 1 | 0.15 | 9 |
| Qatar | 0.08 | 1 | 0.20 | 12 |
| Saint Helena | 0.08 | 1 | 0.15 | 9 |
| Seychelles | 0.08 | 1 | 0.12 | 7 |
| Vanuatu | 0.08 | 1 | 0.13 | 8 |
| Yemen | 0.08 | 1 | 0.08 | 5 |
| American Samoa | 0 | 0 | 0.02 | 1 |
| Anguilla | 0 | 0 | 0.08 | 5 |
| Austria | 0.00 | 0 | 0.05 | 3 |
| Cambodia | 0.00 | 0 | 0.02 | 1 |
| Cameroon | 0.00 | 0 | 0.03 | 2 |
| Cape Verde | 0.00 | 0 | 0.02 | 1 |
| Cook Islands | 0.00 | 0 | 0.02 | 1 |
| Denmark | 0.00 | 0 | 0.08 | 5 |
| Dominican Republic | 0.00 | 0 | 0.02 | 1 |
| Falkland Islands | 0.00 | 0 | 0.02 | 1 |
| Finland | 0.00 | 0 | 0.03 | 2 |
| Gabon | 0.00 | 0 | 0.02 | 1 |
| Grenada | 0.00 | 0 | 0.03 | 2 |
| Guinea | 0.00 | 0 | 0.02 | 1 |
| Ireland | 0.00 | 0 | 0.02 | 1 |
| Jordan | 0.00 | 0 | 0.03 | 2 |
| Kiribati | 0.00 | 0 | 0.10 | 6 |
| Martinique | 0.00 | 0 | 0.03 | 2 |
| Mauritania | 0.00 | 0 | 0.02 | 1 |
| Micronesia | 0.00 | 0 | 0.05 | 3 |
| Monaco | 0.00 | 0 | 0.02 | 1 |
| Morocco | 0.00 | 0 | 0.02 | 1 |
| Nepal | 0.00 | 0 | 0.03 | 2 |
| Palau | 0.00 | 0 | 0.05 | 3 |
| Papua New Guinea | 0.00 | 0 | 0.15 | 9 |
| Poland | 0.00 | 0 | 0.02 | 1 |
| Samoa | 0.00 | 0 | 0.02 | 1 |
| Sao Tome and Principe | 0.00 | 0 | 0.02 | 1 |
| Senegal | 0.00 | 0 | 0.05 | 3 |
| Serbia | 0.00 | 0 | 0.02 | 1 |
| South Korea | 0.00 | 0 | 0.02 | 1 |
| Sudan | 0.00 | 0 | 0.02 | 1 |
| Suriname | 0.00 | 0 | 0.02 | 1 |
| Timor-Leste | 0.00 | 0 | 0.02 | 1 |
| Tonga | 0.00 | 0 | 0.03 | 2 |
| Uruguay | 0.00 | 0 | 0.02 | 1 |
| Zambia | 0.00 | 0 | 0.02 | 1 |
