# Supplementary material for: Tropical marine sciences: Knowledge production in a web of path dependencies
Source: PLoS One. 2020 Feb 6;15(2):e0228613. doi: 10.1371/journal.pone.0228613 (PMC7004553; doi:10.1371/journal.pone.0228613)
Supplement: S2 Table — Categories include the following disciplinary compositions: ‘Natural’ includes ecology, biology, chemistry, geology; ‘Social’ includes political, sociology, anthropology, economics, history; ‘Other’ includes geography, sustainability science other. Data only until 2014 (n = 753). (DOCX) [file pone.0228613.s011.docx]

**Table S2**. The disciplinary focus of articles grouped into categories. Categories include the following disciplinary compositions: ‘Natural’ includes ecology, biology, chemistry, geology; ‘Social’ includes political, sociology, anthropology, economics, history; ‘Other’ includes geography, sustainability science other. Data only until 2014 (n=753).

| **Type of article** | **Disciplinary composition of article** | **Total N** | **Percent of sample** |
| --- | --- | --- | --- |
| Single discipline | Natural science | 314 | 41% |
| Single discipline | Social science | 128 | 16.5% |
| Multiple disciplines | Natural & Natural | 82 | 10.5% |
| Multiple disciplines | Natural & Social | 63 | 8% |
| Multiple disciplines | Natural & Other | 58 | 7.5% |
| Single discipline | Other | 46 | 6% |
| Multiple disciplines | Social & Social | 40 | 5% |
| Multiple disciplines | Social & Other | 34 | 4.5% |
| Multiple disciplines | Other & Other | 7 | 1% |
