# Supplementary material for: Tropical marine sciences: Knowledge production in a web of path dependencies
Source: PLoS One. 2020 Feb 6;15(2):e0228613. doi: 10.1371/journal.pone.0228613 (PMC7004553; doi:10.1371/journal.pone.0228613)
Supplement: S3 Table — Words that were not a noun (“True” in the third column) were removed from the data set, unless they were considered informative (“True” in the fourth column). (DOCX) [file pone.0228613.s012.docx]

**Table S3**. Lemmatization and removal of non-informative nouns, and words, from the most common words of abstracts on marine tropical science, as performed for the cluster analysis to identify assemblages of words that could represent different terminologies (c.f., Methods: *Terminological cluster analysis*). Words that were not a noun (“True” in the third column) were removed from the data set, unless they were considered informative (“True” in the fourth column).

| **Word** | **Synonym group** | **Not a noun** | **Not a noun, but informative** |
| --- | --- | --- | --- |
| **abundance** |  |  |  |
| **action** |  |  |  |
| **activity** |  |  |  |
| **adjacent** |  |  |  |
| **affect** |  |  |  |
| **after** |  |  |  |
| **aim** |  |  |  |
| **along** |  |  |  |
| **although** |  |  |  |
| **analysis** |  |  |  |
| **anthropogenic** |  |  |  |
| **approach** |  |  |  |
| **assemblage** |  |  |  |
| **assess** | **1** |  |  |
| **assessment** | **1** |  |  |
| **australia** |  |  |  |
| **available** |  |  | **True** |
| **barrier** |  |  |  |
| **base** |  |  |  |
| **bay** |  |  |  |
| **benefit** |  |  |  |
| **benthic** |  |  |  |
| **biodiversity** |  |  |  |
| **biological** |  |  |  |
| **biomass** |  |  |  |
| **c** |  | **True** |  |
| **can** |  | **True** |  |
| **caribbean** |  |  |  |
| **case** |  |  |  |
| **catch** |  |  |  |
| **cause** |  |  |  |
| **central** |  |  |  |
| **challenge** |  |  |  |
| **change** |  |  |  |
| **climate** |  |  |  |
| **coast** | **2** |  |  |
| **coastal** | **2** |  |  |
| **community** |  |  |  |
| **compare** |  |  | **True** |
| **complex** |  |  |  |
| **composition** |  |  |  |
| **concentration** |  |  |  |
| **condition** |  |  |  |
| **conservation** |  |  |  |
| **consider** |  |  |  |
| **context** |  |  |  |
| **contribute** |  |  |  |
| **control** |  |  |  |
| **country** |  |  |  |
| **cover** |  |  |  |
| **critical** |  |  |  |
| **current** |  |  |  |
| **datum** |  |  |  |
| **decade** |  |  |  |
| **decline** |  |  |  |
| **degradation** |  |  |  |
| **demonstrate** |  |  |  |
| **density** |  |  |  |
| **depth** |  |  |  |
| **despite** |  |  |  |
| **determine** |  |  |  |
| **develope** | **3** |  |  |
| **development** | **3** |  |  |
| **difference** | **4** |  |  |
| **different** | **4** |  |  |
| **distribution** |  |  |  |
| **diverse** |  |  |  |
| **diversity** |  |  |  |
| **due** |  | **True** |  |
| **during** |  | **True** |  |
| **dynamic** |  |  |  |
| **e** |  | **True** |  |
| **eastern** |  |  | **True** |
| **ecological** |  |  |  |
| **economic** |  |  |  |
| **ecosystem** |  |  |  |
| **effect** | **5** |  |  |
| **effective** | **5** |  |  |
| **effort** |  |  |  |
| **environment** | **6** |  |  |
| **environmental** | **6** |  |  |
| **estimate** |  |  |  |
| **evaluate** |  |  |  |
| **event** |  |  |  |
| **evidence** |  |  |  |
| **examine** |  |  |  |
| **exist** |  |  |  |
| **factor** |  |  |  |
| **far** |  |  |  |
| **few** |  |  |  |
| **find** |  |  |  |
| **fish** |  |  |  |
| **fishery** |  |  |  |
| **focus** |  |  |  |
| **food** |  |  |  |
| **four** |  |  |  |
| **function.** |  |  |  |
| **future** |  |  |  |
| **g** |  | **True** |  |
| **global** |  |  |  |
| **good** |  |  |  |
| **government** |  |  |  |
| **great** |  |  |  |
| **group** |  |  |  |
| **growth** |  |  |  |
| **habitat** |  |  |  |
| **health** |  |  |  |
| **here** |  |  |  |
| **high** | **7** |  |  |
| **highly** | **7** |  |  |
| **however** |  | **True** |  |
| **human** |  |  |  |
| **i** |  | **True** |  |
| **identify** |  |  |  |
| **impact** |  |  |  |
| **importance** | **8** |  |  |
| **important** | **8** |  |  |
| **improve** |  |  |  |
| **include** |  |  |  |
| **increase** |  |  |  |
| **index** |  |  |  |
| **indian** |  |  |  |
| **indicate** |  |  |  |
| **individual** |  |  |  |
| **influence** |  |  |  |
| **information** |  |  |  |
| **investigate** |  |  |  |
| **island** |  |  |  |
| **issue** |  |  |  |
| **key** |  |  |  |
| **km** |  | **True** |  |
| **knowledge** |  |  |  |
| **lagoon** |  |  |  |
| **land** |  |  |  |
| **large** |  |  |  |
| **lead** |  |  |  |
| **level** |  |  |  |
| **likely** |  |  |  |
| **limit** |  |  |  |
| **little** |  |  |  |
| **live** |  |  |  |
| **local** |  |  |  |
| **location** |  |  |  |
| **long.term** |  |  |  |
| **loss** |  |  |  |
| **low** |  |  |  |
| **main** |  |  |  |
| **major** |  |  |  |
| **manage** | **9** |  |  |
| **management** | **9** |  |  |
| **mangrove** |  |  |  |
| **map** |  |  |  |
| **marine** |  |  |  |
| **may** |  |  |  |
| **mean** |  |  |  |
| **measure** |  |  |  |
| **method** |  |  |  |
| **model** |  |  |  |
| **monitor** |  |  |  |
| **national** |  |  |  |
| **natural** |  |  |  |
| **need** |  |  |  |
| **new** |  |  |  |
| **numb** |  |  |  |
| **nutrient** |  |  |  |
| **occur** |  |  |  |
| **ocean** |  |  |  |
| **often** |  |  |  |
| **order** |  |  |  |
| **overall** |  |  |  |
| **pacific** |  |  |  |
| **paper** |  |  |  |
| **park** |  |  |  |
| **part** |  |  |  |
| **pattern** |  |  |  |
| **period** |  |  |  |
| **plan** |  |  |  |
| **policy** |  |  |  |
| **population** |  |  |  |
| **potential** |  |  |  |
| **present** |  |  |  |
| **pressure** |  |  |  |
| **process** |  |  |  |
| **project** |  |  |  |
| **protect** | **11** |  |  |
| **protection** | **11** |  |  |
| **provide** |  |  |  |
| **quality** |  |  |  |
| **range** |  |  |  |
| **rate** |  |  |  |
| **recent** |  |  |  |
| **record** |  |  |  |
| **reduce** |  |  |  |
| **region** | **12** |  |  |
| **regional** | **12** |  |  |
| **relationship** |  |  |  |
| **relative** | **13** |  |  |
| **relatively** | **13** |  |  |
| **remain** |  |  |  |
| **represent** |  |  |  |
| **require** |  |  |  |
| **research** |  |  |  |
| **resource** |  |  |  |
| **response** |  |  |  |
| **result** |  |  |  |
| **reveal** |  |  |  |
| **richness** |  |  |  |
| **role** |  |  |  |
| **sample** |  |  |  |
| **scale** |  |  |  |
| **sea** |  |  |  |
| **seagrass** |  |  |  |
| **sediment** |  |  |  |
| **set** |  |  |  |
| **several** |  |  |  |
| **show** |  |  |  |
| **significant** | **14** |  |  |
| **significantly** | **14** |  |  |
| **similar** |  |  |  |
| **site** |  |  |  |
| **size** |  |  |  |
| **small** |  |  |  |
| **social** |  |  |  |
| **some** |  |  |  |
| **source** |  |  |  |
| **south** | **15** |  |  |
| **southern** | **15** |  |  |
| **spatial** |  |  |  |
| **species** |  |  |  |
| **state** |  |  |  |
| **status** |  |  |  |
| **strategy** |  |  |  |
| **strong** |  |  |  |
| **structure** |  |  |  |
| **suggest** |  |  |  |
| **support** |  |  |  |
| **surface** |  |  |  |
| **survey** |  |  |  |
| **sustainable** |  |  |  |
| **system** |  |  |  |
| **target** |  |  |  |
| **temperature** |  |  |  |
| **term** |  |  |  |
| **there** |  |  |  |
| **threat** |  |  |  |
| **throughout** |  |  |  |
| **time** |  |  |  |
| **tool** |  |  |  |
| **total** |  |  |  |
| **tourism** |  |  |  |
| **tropical** |  |  |  |
| **two** |  |  |  |
| **type** |  |  |  |
| **under** |  |  |  |
| **understand** |  |  |  |
| **use** |  |  |  |
| **value** |  |  |  |
| **variability** |  |  |  |
| **variable** |  |  |  |
| **variation** |  |  |  |
| **water** |  |  |  |
| **well** |  |  |  |
| **western** |  |  |  |
| **when** |  |  |  |
| **world** |  |  |  |
| **year** |  |  |  |
| **zone** |  |  |  |
| **?** |  | **True** |  |
| **2015** |  |  |  |
| **2017** |  |  |  |
| **2018** |  |  |  |
| **5** |  |  |  |
