# Supplementary material for: Tropical marine sciences: Knowledge production in a web of path dependencies
Source: PLoS One. 2020 Feb 6;15(2):e0228613. doi: 10.1371/journal.pone.0228613 (PMC7004553; doi:10.1371/journal.pone.0228613)
Supplement: S4 Table — (DOCX) [file pone.0228613.s013.docx]

**Table S4.** Step-by-step systematic review procedure.

| Steps | Procedure | Results |
| --- | --- | --- |
| 1) Data gathering | Search Scopus database using defined search string with indicator words to enable the inclusion of all potentially relevant articles. | 1,995 articles |
| 2) Data screening | Title scan of all initial 1,995 articles for broad relevance. | Crude elimination of articles outside of scope |
| 3) Data cleaning | Apply three specific selection criteria to each article through reading abstract and/or full text scan.  1) Within or directly related to the tropics.  2) Within or directly related to the marine environment.  3) Focus on processes directly related to social, ecological or social-ecological systems. | Fine elimination of articles outside of scope |
| 4) Data scoping | Download of all papers classified as potentially relevant. | 753 articles available for data collection |
| 5) Review category development | Collaboratively decide review categories to assess each paper by. Develop uniform data collection sheet for each article and predefined definitions for each category. | 13 defined review categories. |
| 6) Article review | Collect data through reading title, abstract and text of each article based on collectively decided review categories. Submit data through collection form into database. | Collect data on all 753 articles through review categories. |
