# Supplementary material for: Tropical marine sciences: Knowledge production in a web of path dependencies
Source: PLoS One. 2020 Feb 6;15(2):e0228613. doi: 10.1371/journal.pone.0228613 (PMC7004553; doi:10.1371/journal.pone.0228613)
Supplement: S5 Table — (DOCX) [file pone.0228613.s014.docx]

**Table S5.** Node (country) centrality metrics for the undirected authorship network.

| **Country** | **Degree** | **Strength** | **Closeness** | **Betweenness** | **Eigen** |
| --- | --- | --- | --- | --- | --- |
| American Samoa | 28 | 64 | 0.0014793 | 19.86544577 | 0.02453 |
| Anguilla | 2 | 40 | 0.0003332 | 0 | 0.015662 |
| Argentina | 2 | 12 | 0.0006878 | 0 | 0.000987 |
| Australia | 136 | 3416 | 0.0015038 | 824.4647397 | 0.912635 |
| Austria | 12 | 46 | 0.0012422 | 24.95446461 | 0.013269 |
| Bahamas | 28 | 160 | 0.0013351 | 27.5124635 | 0.060218 |
| Bahrain | 2 | 8 | 0.0008489 | 0 | 8.16E-05 |
| Bangladesh | 8 | 52 | 0.000956 | 0 | 0.005608 |
| Barbados | 34 | 140 | 0.0014006 | 86.32286511 | 0.032185 |
| Belgium | 46 | 396 | 0.0013812 | 283.6892781 | 0.106906 |
| Belize | 2 | 68 | 0.0002074 | 0 | 0.042198 |
| Benin | 6 | 28 | 0.001105 | 0 | 0.008109 |
| Bermuda | 4 | 64 | 0.0005711 | 0 | 0.012731 |
| Brazil | 38 | 426 | 0.0013405 | 361.1714728 | 0.129609 |
| Brunei | 8 | 14 | 0.0014306 | 22.7836557 | 0.0027 |
| Cambodia | 6 | 24 | 0.0012376 | 56.5139442 | 0.003363 |
| Cameroon | 2 | 16 | 0.0005995 | 0 | 0.001088 |
| Canada | 90 | 860 | 0.0016 | 1325.04067 | 0.306134 |
| Cape Verde | 28 | 64 | 0.0014706 | 19.86544577 | 0.02453 |
| Chile | 14 | 48 | 0.0012953 | 1.131182796 | 0.014735 |
| China | 40 | 316 | 0.0014793 | 253.6897447 | 0.078514 |
| Colombia | 40 | 176 | 0.0014104 | 318.8223362 | 0.042802 |
| Cook Islands | 8 | 18 | 0.0013158 | 49.60996067 | 0.005024 |
| Costa Rica | 30 | 282 | 0.0012077 | 8.010790407 | 0.07333 |
| Cuba | 36 | 234 | 0.001292 | 61.02179256 | 0.050235 |
| Czech Republic | 6 | 20 | 0.0012346 | 0 | 0.000719 |
| Denmark | 36 | 98 | 0.0014493 | 59.12641273 | 0.029415 |
| Dominican Republic | 22 | 44 | 0.001385 | 150.3366494 | 0.004792 |
| Ecuador | 24 | 142 | 0.0013055 | 5.269026888 | 0.023466 |
| Egypt | 26 | 80 | 0.0015198 | 183.2301286 | 0.007582 |
| El Salvador | 4 | 36 | 0.0005208 | 0 | 0.012728 |
| Falkland Islands | 4 | 28 | 0.0006361 | 0 | 0.006605 |
| Fiji | 16 | 208 | 0.001328 | 55.84411604 | 0.108912 |
| Finland | 2 | 4 | 0.0010787 | 0 | 0.000126 |
| France | 112 | 1506 | 0.0014045 | 288.378132 | 0.319527 |
| French Guiana | 4 | 8 | 0.001328 | 0 | 0.002016 |
| French Polynesia | 28 | 162 | 0.001506 | 125.2945601 | 0.035768 |
| Gabon | 28 | 64 | 0.0014684 | 19.86544577 | 0.02453 |
| Germany | 78 | 1050 | 0.0014104 | 258.4832433 | 0.277254 |
| Ghana | 28 | 76 | 0.0014684 | 19.86544577 | 0.031964 |
| Grenada | 2 | 16 | 0.0005708 | 0 | 0.009944 |
| Guam | 10 | 22 | 0.0014265 | 210.8191994 | 0.009986 |
| Guinea | 28 | 64 | 0.0014684 | 19.86544577 | 0.02453 |
| Hong Kong | 40 | 132 | 0.0015674 | 789.7902477 | 0.02038 |
| India | 36 | 194 | 0.001462 | 211.0319053 | 0.057565 |
| Indonesia | 58 | 678 | 0.0015898 | 464.6466315 | 0.217619 |
| Iran | 4 | 28 | 0.000668 | 0 | 0.010754 |
| Ireland | 2 | 2 | 0.001221 | 0 | 3.17E-05 |
| Israel | 10 | 24 | 0.0013889 | 199.0086946 | 0.003793 |
| Italy | 52 | 332 | 0.0014368 | 103.8039286 | 0.06459 |
| Ivory Coast | 2 | 10 | 0.000768 | 0 | 0.002012 |
| Jamaica | 28 | 96 | 0.0012739 | 2.757483393 | 0.01118 |
| Japan | 58 | 278 | 0.0015601 | 498.3492346 | 0.048126 |
| Jordan | 4 | 12 | 0.0009569 | 0 | 0.00254 |
| Kenya | 36 | 370 | 0.0014085 | 240.0733342 | 0.126562 |
| Kiribati | 10 | 204 | 0.0007686 | 0 | 0.107014 |
| La Reunion | 12 | 56 | 0.0014903 | 170.6319777 | 0.010001 |
| Madagascar | 8 | 92 | 0.001105 | 0 | 0.03822 |
| Malaysia | 50 | 736 | 0.0013755 | 326.9943729 | 0.234041 |
| Maldives | 14 | 72 | 0.0010787 | 0 | 0.02915 |
| Martinique | 2 | 12 | 0.0007418 | 0 | 0.004712 |
| Mauritania | 28 | 64 | 0.0014684 | 19.86544577 | 0.02453 |
| Mauritius | 16 | 54 | 0.0012531 | 1.305555556 | 0.008786 |
| Mexico | 58 | 782 | 0.0014286 | 336.4543133 | 0.224826 |
| Micronesia | 8 | 42 | 0.0011249 | 0 | 0.016108 |
| Monaco | 2 | 10 | 0.0007168 | 0 | 0.006222 |
| Morocco | 10 | 20 | 0.0012821 | 1.71014911 | 0.002528 |
| Mozambique | 10 | 54 | 0.0011481 | 0 | 0.016524 |
| Namibia | 0 | 0 | 6.887E-05 | 0 | 0.240863 |
| Nepal | 8 | 24 | 0.0013193 | 16.89938247 | 0.010179 |
| Netherlands | 62 | 544 | 0.0014535 | 251.660896 | 0.135536 |
| Netherlands Antilles | 24 | 166 | 0.0010438 | 0 | 0.025379 |
| New Caledonia | 46 | 430 | 0.0014388 | 333.6540626 | 0.130884 |
| New Zealand | 26 | 176 | 0.0014535 | 64.70407948 | 0.063471 |
| Nicaragua | 14 | 100 | 0.0010194 | 109.3350892 | 0.028377 |
| Nigeria | 28 | 64 | 0.0014684 | 19.86544577 | 0.02453 |
| Norway | 10 | 28 | 0.0011249 | 179.6846655 | 0.004064 |
| Oman | 8 | 40 | 0.0011377 | 28.88306337 | 0.012653 |
| Pakistan | 6 | 12 | 0.0012739 | 4.981818182 | 0.005105 |
| Palau | 8 | 42 | 0.000993 | 0 | 0.014774 |
| Panama | 20 | 248 | 0.0013158 | 41.93235665 | 0.093017 |
| Papua New Guinea | 20 | 118 | 0.0014265 | 42.17429323 | 0.044921 |
| Peru | 4 | 74 | 0.0007148 | 0 | 0.018998 |
| Philippines | 56 | 844 | 0.0014663 | 343.417923 | 0.288066 |
| Poland | 4 | 8 | 0.0013263 | 0 | 0.000837 |
| Portugal | 30 | 150 | 0.0013793 | 160.8977871 | 0.035072 |
| Puerto Rico | 28 | 88 | 0.0012937 | 136.5846834 | 0.026505 |
| Qatar | 14 | 110 | 0.001385 | 42.8269833 | 0.042823 |
| Russia | 10 | 68 | 0.001 | 0 | 0.002807 |
| Saint Helena | 8 | 144 | 0.0007174 | 0 | 0.041565 |
| Samoa | 10 | 44 | 0.0013986 | 90.88309529 | 0.018684 |
| Sao Tome and Principe | 2 | 8 | 0.0008354 | 0 | 0.000197 |
| Saudi Arabia | 44 | 232 | 0.0013369 | 3.593293727 | 0.044783 |
| Senegal | 32 | 92 | 0.0014286 | 125.8401601 | 0.027331 |
| Serbia | 4 | 12 | 0.0007634 | 0 | 0.001491 |
| Seychelles | 16 | 52 | 0.0012755 | 232.6163606 | 0.014383 |
| Singapore | 40 | 510 | 0.0014124 | 189.2518758 | 0.074081 |
| Solomon Islands | 22 | 84 | 0.0014793 | 191.0626947 | 0.036023 |
| South Africa | 66 | 310 | 0.0014903 | 387.9000417 | 0.107769 |
| South Korea | 4 | 12 | 0.0007634 | 0 | 0.001491 |
| Spain | 52 | 504 | 0.001443 | 477.5526489 | 0.118941 |
| Sri Lanka | 12 | 58 | 0.0012987 | 0.363408521 | 0.011571 |
| Sudan | 8 | 14 | 0.0013831 | 60.04772132 | 0.003143 |
| Suriname | 2 | 8 | 0.0008285 | 0 | 0.000554 |
| Sweden | 40 | 302 | 0.0013947 | 46.72864516 | 0.094099 |
| Switzerland | 44 | 206 | 0.0014903 | 107.0274575 | 0.055056 |
| Taiwan | 28 | 158 | 0.0012361 | 10.34928788 | 0.029445 |
| Tanzania | 28 | 158 | 0.0014327 | 234.2608069 | 0.042406 |
| Thailand | 42 | 306 | 0.0013812 | 64.9972653 | 0.066786 |
| Timor-Leste | 14 | 20 | 0.0014837 | 318.8000121 | 0.006362 |
| Tonga | 10 | 22 | 0.0013532 | 69.57010912 | 0.008484 |
| Trinidad and Tobago | 24 | 126 | 0.0010917 | 0 | 0.014965 |
| UAE | 20 | 46 | 0.0014184 | 340.8950222 | 0.012004 |
| UK | 126 | 2232 | 0.0015649 | 1255.797845 | 0.630792 |
| Uruguay | 2 | 6 | 0.0009099 | 0 | 0.000503 |
| USA | 172 | 5286 | 0.0012438 | 874.1189416 | 1 |
| Vanuatu | 12 | 66 | 0.0012723 | 7.82026862 | 0.02105 |
| Venezuela | 22 | 120 | 0.0010081 | 0 | 0.013817 |
| Vietnam | 30 | 156 | 0.0013605 | 25.99005736 | 0.015746 |
| Yemen | 6 | 16 | 0.0011468 | 0 | 0.001668 |
| Zambia | 6 | 10 | 0.001328 | 13.13625198 | 0.000545 |
