# Supplementary material for: Tropical marine sciences: Knowledge production in a web of path dependencies
Source: PLoS One. 2020 Feb 6;15(2):e0228613. doi: 10.1371/journal.pone.0228613 (PMC7004553; doi:10.1371/journal.pone.0228613)
Supplement: S6 Table — (DOCX) [file pone.0228613.s015.docx]

**Table S6.** Network metric definitions.

| **Metric** | **Definition (how measured)** |
| --- | --- |
| Eigenvector centrality | a measure of being well-connected, or connected to the well-connected. Only works on undirected networks. |
| Strength | a weighted measure of degree that takes into account the number of edges that go from one node to another. |
| Betweenness | Approximately the number of shortest paths between nodes that pass through a particular node. |
| Closeness | how many edges are required to access every other node from a given node. Distance measure of how many edges to arrive at another node. Higher values mean less centrality. |
| Degree | the number of adjacent edges to each node. Weighted includes total number. Unweighted only includes unique edges. In-degree only measures the number of edges coming into a node in a directed network. |
| Network diameter | he length of the longest path (in number of edges) between two nodes. |
| Network mean distance | the average number of edges between any two nodes in the network. |
