# Supplementary material for: Tropical marine sciences: Knowledge production in a web of path dependencies
Source: PLoS One. 2020 Feb 6;15(2):e0228613. doi: 10.1371/journal.pone.0228613 (PMC7004553; doi:10.1371/journal.pone.0228613)
Supplement: S7 Table — (DOCX) [file pone.0228613.s016.docx]

**Table S7.** Unique collaborations with other countries.

| **Country** | **Unique collaborations with other countries** |
| --- | --- |
| USA | 86 |
| Australia | 68 |
| UK | 63 |
| France | 56 |
| Canada | 45 |
| Germany | 39 |
| South Africa | 33 |
| Netherlands | 31 |
| Indonesia | 29 |
| Japan | 29 |
| Mexico | 29 |
| Philippines | 28 |
| Italy | 26 |
| Spain | 26 |
| Malaysia | 25 |
| Belgium | 23 |
| New Caledonia | 23 |
| Saudi Arabia | 22 |
| Switzerland | 22 |
| Thailand | 21 |
| China | 20 |
| Colombia | 20 |
| Hong Kong | 20 |
| Singapore | 20 |
| Sweden | 20 |
| Brazil | 19 |
| Cuba | 18 |
| Denmark | 18 |
| India | 18 |
| Kenya | 18 |
| Barbados | 17 |
| Senegal | 16 |
| Costa Rica | 15 |
| Portugal | 15 |
| Vietnam | 15 |
| American Samoa | 14 |
| Bahamas | 14 |
| Cape Verde | 14 |
| French Polynesia | 14 |
| Gabon | 14 |
| Ghana | 14 |
| Guinea | 14 |
| Jamaica | 14 |
| Mauritania | 14 |
| Nigeria | 14 |
| Puerto Rico | 14 |
| Taiwan | 14 |
| Tanzania | 14 |
| Egypt | 13 |
| New Zealand | 13 |
| Ecuador | 12 |
| Netherlands Antilles | 12 |
| Trinidad and Tobago | 12 |
| Dominican Republic | 11 |
| Solomon Islands | 11 |
| Venezuela | 11 |
| Panama | 10 |
| Papua New Guinea | 10 |
| UAE | 10 |
| Fiji | 8 |
| Mauritius | 8 |
| Seychelles | 8 |
| Chile | 7 |
| Maldives | 7 |
| Nicaragua | 7 |
| Qatar | 7 |
| Timor-Leste | 7 |
| Austria | 6 |
| La Reunion | 6 |
| Sri Lanka | 6 |
| Vanuatu | 6 |
| Guam | 5 |
| Israel | 5 |
| Kiribati | 5 |
| Morocco | 5 |
| Mozambique | 5 |
| Norway | 5 |
| Russia | 5 |
| Samoa | 5 |
| Tonga | 5 |
| Bangladesh | 4 |
| Brunei | 4 |
| Cook Islands | 4 |
| Madagascar | 4 |
| Micronesia | 4 |
| Nepal | 4 |
| Oman | 4 |
| Palau | 4 |
| Saint Helena | 4 |
| Sudan | 4 |
| Benin | 3 |
| Cambodia | 3 |
| Czech Republic | 3 |
| Pakistan | 3 |
| Yemen | 3 |
| Zambia | 3 |
| Bermuda | 2 |
| El Salvador | 2 |
| Falkland Islands | 2 |
| French Guiana | 2 |
| Iran | 2 |
| Jordan | 2 |
| Peru | 2 |
| Poland | 2 |
| Serbia | 2 |
| South Korea | 2 |
| Anguilla | 1 |
| Argentina | 1 |
| Bahrain | 1 |
| Belize | 1 |
| Cameroon | 1 |
| Finland | 1 |
| Grenada | 1 |
| Ireland | 1 |
| Ivory Coast | 1 |
| Martinique | 1 |
| Monaco | 1 |
| Sao Tome and Principe | 1 |
| Suriname | 1 |
| Uruguay | 1 |
