# Supplementary material for: Tropical marine sciences: Knowledge production in a web of path dependencies
Source: PLoS One. 2020 Feb 6;15(2):e0228613. doi: 10.1371/journal.pone.0228613 (PMC7004553; doi:10.1371/journal.pone.0228613)
Supplement: S8 Table — (DOCX) [file pone.0228613.s017.docx]

**Table S8.** Linear regression model

| Residuals:  Min 1Q Median 3Q Max  -4.1570 -0.7757 0.1515 0.8389 2.9484  Coefficients:  Estimate Std. Error t value Pr(>\|t\|)  (Intercept) 3.7088 0.1130 32.829 < 2e-16 ***  Ratio_perc 1.5468 0.1679 9.211 1.39e-15 ***  ---  Signif. codes: 0 ‘***’ 0.001 ‘**’ 0.01 ‘*’ 0.05 ‘.’ 0.1 ‘ ’ 1  Residual standard error: 1.167 on 119 degrees of freedom  Multiple R-squared: 0.4162, Adjusted R-squared: 0.4113  F-statistic: 84.84 on 1 and 119 DF, p-value: 1.385e-15 |
| --- |
